# Supplementary material for: Salicylic acid metabolism and signalling coordinate senescence initiation in aspen in nature
Source: Nat Commun. 2023 Jul 18;14:4288. doi: 10.1038/s41467-023-39564-5 (PMC10354028; doi:10.1038/s41467-023-39564-5)

Supplementary Materials for

**Salicylic Acid Metabolism and Signalling Coordinate Senescence Initiation in Aspen in Nature**

Jenna Lihavainen, Jan Šimura, Pushan Bag, Nazeer Fataftah, Kathryn Megan Robinson, Nicolas Delhomme, Ondřej Novák, Karin Ljung, Stefan Jansson^*^

*Corresponding author. Email: stefan.jansson[@umu.se](mailto:xxxxx@xxxx.xxx)

**This PDF file includes:**

Supplementary Methods 1 and 2

Figures S1 to S25

Table S1

Supplementary Note 1 and 2

**Other Supplementary Materials for this manuscript include the following:**

Supplementary Data 1 to 16

Other source data files/folders:

Source Data 1

Source Data 2

Source Data 3

Source Data 4

**Supplementary Methods 1**

**Phytohormones**

Phytohormone analyses were performed for the leaf samples collected at eleven time points from five SwAsp genotypes in autumn 2018 (n=2-3 individual trees per genotype and time point). Leaf samples (~10 mg FW) were extracted with 1 mL of ice cold 10% MeOH with 1 M formic acid (FA). Labelled internal standards (10 µL) and two tungsten beads (3 mm diameter) were added to each sample and samples were homogenized for 10 min in a bead mill (25 Hz, Retsch Qiagen). Samples were centrifuged for 15 min at 18 000 ×g at 4 °C. The supernatant was divided into two 450 µL aliquots that were each diluted to 1 mL with 1 M formic acid (FA) and with water for analysis of cytokinins (CKs) and stress induced phytohormones (SIP, jasmonic acid, auxin, salicylic acid, abscisic acid and related metabolites), respectively. The purification of isoprenoid CKs was carried out according to Dobrev and Kaminek ^1^ using the Oasis MCX column (30 mg of C18/SCX combined sorbent with cation-exchange properties, Waters Inc., Milford, MA, USA). Analytes were eluted by two-step elution using a 0.35 M NH_4_OH aqueous solution and 0.35 M NH_4_OH in 60% (v/v) MeOH solution and the other set of samples for SIP analysis was purified using the Oasis HLB (30 mg 1 cc, Waters Inc., Milford, MA, USA) as described in Floková et al. ^2^. The eluates were collected in test tubes, evaporated to dryness using a speed vac (SpeedVac SPD111V, Thermo Scientific, Waltham, MA, USA) and stored at -70 °C.

Prior to analysis, samples were dissolved in 40 µL of 30% MeOH and analysed with the UPLC-ESI-MS/MS system comprising of a 1290 Infinity Binary LC System coupled to a 6490 Triple Quad LC/MS System with Jet Stream and Dual Ion Funnel technologies (Agilent Technologies, Santa Clara, CA, USA). The UPLC-ESI-MS/MS parameters used to analyse CK and SIP levels were described in Svačinová et al. ^3^ and Floková et al. ^2^, respectively. Phytohormone levels were quantified by normalizing analyte peak area by labelled internal standard peak area, using a calibration curve of the reference standard and the fresh weight of the sample using Agilent MassHunter Workstation Software Quantitative (Agilent Technologies, Santa Clara, CA, USA). The concentrations of detected 23 phytohormones were expressed as pmol g^-1^ FW.

**GC-MS metabolomics**

Metabolite analyses were performed with the same SwAsp samples as for phytohormones, and with the leaf samples of genotype 201 from twelve time points during the autumn. Soluble metabolites were extracted from the leaf samples (~10 mg FW) with 1 mL of cold MeOH:H_2_O:chloroform (3:1:1), containing internal standards (proline-^13^C_5_, succinic acid-d_4_, alpha-ketoglutarate-^13^C_4_, glutamic acid-^13^C_5_, putrescine-d_4_, D-glucose-^13^C_6_, sucrose-^13^C_12_, myristic acid-^13^C_3_, hexadecanoic acid-^13^C_4_, cholesterol-d_7_, salicylic acid-d_4_). Two tungsten beads were added to each sample and the samples were homogenized for 3 min in a bead mill (27 Hz, Retsch GmHG) followed by extraction for 5 min in a vortex mixer. Samples were centrifuged for 10 min at 18 000 ×g at 4 °C. Quality control (QC) samples combined from aliquots from each genotype and time point and alkane series (C7-C40) were included in each GC-MS analysis batch. An aliquot (200 µL) of the supernatant was transferred into a vial and dried using a speed vac and stored at -70 °C.

Samples were derivatized for GC-MS analysis as described in Gullberg et al. ^4^. QC samples and alkane series (C7-C40) were included in each GC-MS analysis batch. The GC-MS system consisted of a GC PAL autosampler (CTC Analytics) combined with a column oven (7890A Agilent Technologies) and Pegasus HT GC-MS/QTOF (LECO) with electron ionization of 70 eV. Sample (1 µL) was injected in 20:1 split mode into a metal liner with deactivated wool (5.2 × 6.3 × 78.5 mm, Restek). Inlet temperature was set to 260 °C and helium flow in the column (J&W DB-5MS Ultra Inert 30 m, ⌀ 0.25 mm, 0.25 µm film thickness, Agilent Technologies) was kept constant at 1 mL min^-1^. The temperature of the column was kept at 70 °C for 2 min, and then increased by 30 °C min^-1^ to 200 °C, by 5 °C min^-1^ to 220 °C, and by 15 °C min^-1^ to 320 °C, where the temperature was kept for 4 min. Transfer line and source temperatures were 270 °C and 200 °C, respectively. Scans were recorded with a rate of 20 spectra s^-1^ in a mass range of 50-800 m/z.

In-house software (SMC_RDA, Swedish Metabolomics Centre) was used to process the GC-MS data as netCDF files. Metabolite peak areas were normalized with the peak area of internal standard, succinic acid-d_4_, and sample weight. All subsequent statistics were performed with normalized data. Metabolites were annotated based on retention index (RI) and mass spectra compared to in-house library (Swedish Metabolomics Centre) and databases (NIST, Golm metabolome database GMD, Fiehn library).

**Supplementary Methods 2**

**Weighted gene co-expression network analysis (WGCNA)**

A weighted gene co-expression network analysis was performed with the WGCNA package (version 1.69) in R (version 4.0.0) as described by Langfelder and Horvath^5^. WGCNA was performed separately for the 2018 and 2011 data sets. Since the consensus network analysis is based on merging the results of many networks, we decided to perform WGCNA separately for 2018 and 2011 data sets to reduce the contribution of the 2011 data set that is of lower quality with less replication, samples and gene coverage than of the 2018 data. For the 2018 data, the analysis included 76 samples (Fig. S5a). First, we filtered out low count genes that were not expressed in at least 50 % of the samples (in 38 out of 76 samples). Next, genes were filtered out if they were not expressed in all three genotypes in at least in one time point. Last, genes that showed low variation in their expression over the time course with coefficient of variation of less than 0.1 were filtered out. At the end, the WGCNA was performed with 21602 genes. Genotypic networks (E81, I48 and L1) and a consensus network were generated using the blockwiseModules function with a soft threshold power of 9 (Fig. S5b). A topological overlap matrix (TOM) was constructed from the adjacency matrix using signed Pearson correlations. Gene modules were defined based on a dynamic tree algorithm using height of 0.15, deep split of 3, minimum module size of 50 and minimum module membership to stay 0.1 (Fig. S5c). Analysis of the 2011 data set was performed with 11 time points (one outlier sample collected on 258 DOY after senescence onset was excluded) and 19287 genes using a soft threshold power of 5 (Fig. S12). The details of the results and the lists of genes assigned in each module are in Supplementary Data 3 (2018) and 5 (2011).

**Module preservation**

Differential analysis (Z summary statistics, Langfelder et al. 2011) was performed to investigate the preservation of the eigengene modules and the module relationships between the three SwAsp genotypes in 2018 (Fig. S6). Perturbation in a gene module means that the gene-to-gene relationships are not conserved in all three genotypes, and the perturbed modules are likely to affect the information flow through the network structure (i.e. the regulatory network has been differently re-wired in the genotypes). Most of the modules were highly preserved (D ~0.9) in the three genotypes. One module (XX) out of 27 showed slightly lower preservation (D <0.9) in genotypes with earlier senescence onset (E81 and I48) compared to L1 that senesced later in the autumn (Fig. S6). Two other smaller modules (XXI and XXII) were less preserved in I48 compared to L1 and E81 (Fig. S6).

**Correlation of eigengene modules with weather parameters, chlorophyll content index and phytohormone levels**

Eigengenes represent the first principal component (PC) and essentially the signature expression of the module. The eigengene patterns detected in 2018 and 2011 are in Fig. 3, Fig. S11b (2018) and Fig. S13 (2011). Correlation of eigengene patterns and individual genes with weather parameters (Fig. S8), with chlorophyll content index (CCI) and metabolites (phytohormones, GC-MS metabolites, other metabolic markers) were studied in both study years (Figs. S10-S11, Fig. S14, Supplementary Data 7-13). In 2018, relationships were studied separately in each genotype and in a consensus network (Fig. S8, Figs. S10-S11). In all cases, *P*-value <0.05 was considered significant. Since the sampling of leaves was performed at noon, weather parameters at the time of sampling and over the past 12 and 24 hours were used for analysis.

In 2018, the relationships between gene expression and weather parameters were highly consistent across the three SwAsp genotypes and persisted in the consensus network (Fig. S10, Fig. S11d). Several modules showed significant correlation with air temperature parameters, relative air humidity (RH%), vapour pressure deficit (VPD) and maximum solar radiation (Fig. S8, Fig. S11d). The results of correlation analyses of two years were compared to identify the major sources of variation in the transcriptome data and genes that exhibited consistent correlations with weather parameters and chlorophyll content index (CCI) irrespective of genotype or study year (Fig. S15).

The correlation between gene expression and CCI were dependent on the genotype (Fig. S10, Fig. S15). In E81, I48 and I201, a large number of individual genes as well as the largest modules with gradually up- and downregulated expression patterns during autumn showed significant correlation with CCI (Fig. S10, Fig. S14). However, such a relationship was absent in L1, in which the expression of genes changed but chlorophyll levels did not (Fig. S15).

Although some of the significant correlations between the modules and cytokinin (CK) and auxin (IAA) metabolites persisted in a consensus network (Fig. S10), most of the relationships between gene expression and phytohormone levels were strongly dependent on the SwAsp genotype (Fig. S11d). The largest gene modules with gradually up- (module II) and down-regulated (modules I and III) expression and senescence associated genes (SAGs) correlated significantly and consistently with CK and IAA metabolite levels in all three SwAsp genotypes (Fig. 3, Fig. S10, Fig. S11). Overall, the stress-induced phytohormones, abscisic acid (ABA), jasmonic acid (JA) and salicylic acid (SA), displayed considerable variation in their association with gene expression patterns (modules and individual genes) between the studied SwAsp genotypes (Fig. S10, Fig. S11d, Supplementary Data 15). Nevertheless, SA levels showed significant positive correlation with the module containing genes involved in SA-mediated signalling pathway (module X) in genotypes E81 (Pearson *r*=0.63, *P*-value=0.0008, two-sided) and I48 (*r*=0.55, *P*-value=0.002), while the correlation was weaker in L1 (*r*=0.36, *P*-value=0.06, two-sided, Fig. S10). In addition, SA levels showed significant positive correlation with the module containing genes involved in endoplasmic reticulum (ER) stress (module XVIII) in genotypes I48 (*r*=0.69, *P*-value=4.0e-5) and L1 (*r*=0.58, *P*-value=0.002) (Fig. S10), whereas the correlation was weaker in E81 (*r*=0.32, *P*-value=0.1, two-sided, Fig. S10). However, individual ER stress- and UPR-related genes correlated positively with SA levels in all three SwAsp genotypes (Fig. 5, Fig. S20). Overall, the ER stress-related module showed different connectivity with other modules, weather parameters and phytohormone levels between the three SwAsp genotypes in autumn 2018 (Fig. S6, Fig. S8-S11).

**Screening of hub genes and Gene Ontology (GO) terms associated with gene modules**

Genes (nodes) with high intra-modular connectivity (module membership, MM) were considered as the primary hub genes that are candidate regulators of biological processes. The top 20 hub genes in the gene modules in the 2018 and 2011 networks are listed in Supplementary Data 4 and 6, respectively. Gene Ontology (GO) enrichment analyses were performed to identify biological processes (BP), molecular functions (MF) and cellular components (CC) enriched in the modules. GO enrichment analyses were performed with *Populus tremula* gene IDs (Potra IDs) with PlantGenIE (<http://plantgenie.org>) ^6^ and complemented with similar analysis for enriched biological processes using best DIAMOND hits for Arabidopsis (ATG ID). Kyoto Encyclopedia of Genes and Genomes (KEGG) pathway enrichment analysis was performed with g:Profiler (<https://biit.cs.ut.ee/gprofiler/gost>) ^7^ with ATG IDs. In most cases, the hub genes were involved in the GO terms and KEGG pathways that were enriched in the module. The results of GO term and KEGG pathway enrichment analyses are in Supplementary Data 4 (2018) and 6 (2011).

**Gene co-expression network visualisation**

Co-expression networks were produced for each genotype separately and then merged (intersected) in Cytoscape (version 3.8.0) ^8^ to identify consistent relationships (edges with FDR adjusted *P*-value <0.05 two-sided) between genes, eigengenes, weather parameters and metabolite levels. GO term networks for different gene sets (SAGs, SA-responsive genes) were produced with ClueGO (2.5.7) ^9^ and CluePedia (version 1.5.7) ^10^ applications in Cytoscape using best DIAMOND hits for ATG IDs (obtained from PlantGenIE) to investigate the connections between biological processes in autumn. GO terms with FDR adjusted *P*-value <0.05 (two-sided) were included in the visualisation. The close neighbours of 135 SA-responsive genes in all three SwAsp genotypes were visualised in a co-expression network. Edges with Pearson correlation coefficient *r* > 0.6 (positive relationships) were included in the network and genes with consistent correlation with SA levels in all three SwAsp genotypes were represented as diamond shapes (Fig. S17). Co-expression networks were visualised with an organic layout so that the size of the node is proportional to its degree (connectivity), larger size depicting higher degree. Nodes with high degree are considered as important hubs with potential regulatory role and nodes with high betweenness centrality as important mediators controlling the information flow through the network structure. In the module networks, a hierarchical layout was used, and eigengenes, weather parameters and metabolites (chlorophyll and phytohormones) were represented as circle, square and diamond shapes, respectively (Fig. 3, Fig. S11, Fig. S13).

**Senescence-associated genes (SAGs) in Populus spp.**

Genes with enhanced or repressed expression during autumn in all four studied aspen genotypes were considered as senescence-associated genes (SAGs, FDR adjusted *P*-value <0.01 two-sided, log_2_ fold change >1.0 between 217-265 DOY in 2011 and 225-264 DOY in 2018). Consistent SAGs irrespective of genotype or study year were identified by comparing the gene lists obtained for each aspen genotype (Venn diagram, Fig. 2). Aspen SAGs were compared with two data sets of SAGs identified in poplar (*P.trichocarpa,* LSD 3.0, <https://bigd.big.ac.cn/lsd/poplar.php>) ^11,12^. For comparison of the data sets from two species, *Populus tremula* gene IDs (Potra gene ID) were converted to the best DIAMOND hit for poplar gene IDs (Potri gene ID) obtained from PlantGenIE. For the identification of enriched GO terms within consistent senescence-associated genes (SAGs) in aspen (*P. tremula*) and poplar (*P.trichocarpa*), best DIAMOND hits for ATG IDs (obtained from PlantGenIE) were used to construct GO term networks as described above. GO terms with FDR adjusted *P*-value <0.05 containing consistent SAGs in both *Populus* species were included in the visualisation (Fig. 2c). In addition, PFAM (protein family and domain) enrichment analyses were performed with PlantGenIE for genes identified as SAGs in both *Populus* spp. using *P.trichocarpa* gene IDs (Potri IDs). The lists of identified SAGs, their correlation with weather parameters and metabolite markers (phytohormone levels, chlorophyll content index), and results of PFAM enrichment analysis are in Supplementary Data 2.

**Supplementary Figures**

**
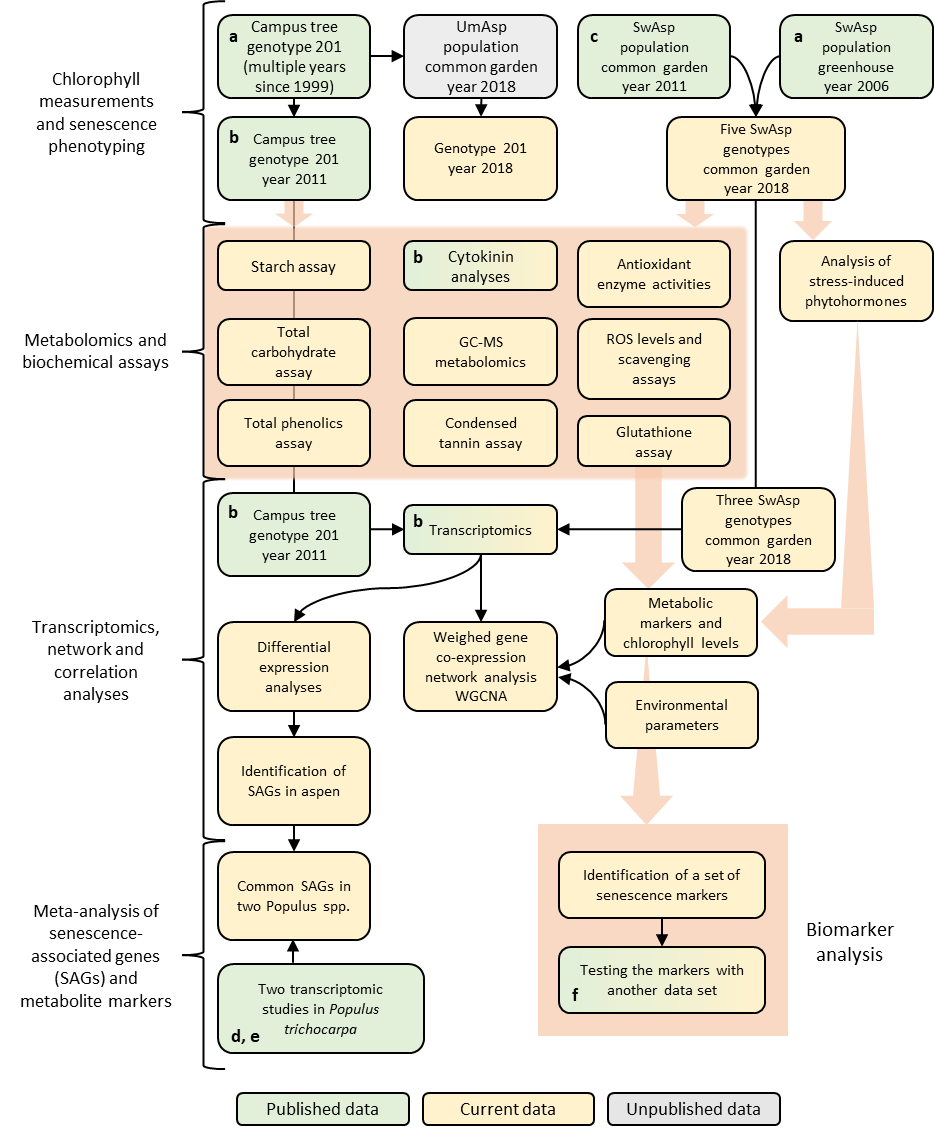
**

**Fig. S1.** Overview of the analyses conducted in this manuscript and the sources of the previously published data integrated with the data analyses. **a**) Fracheboud et al.^13^ , **b**) Edlund et al. ^14^ , **c**) Michelson et al. ^15^ , **d**) Li et al. ^11^, **e**) Lu et al.,^12^ , **f**) Lihavainen et al. ^16^


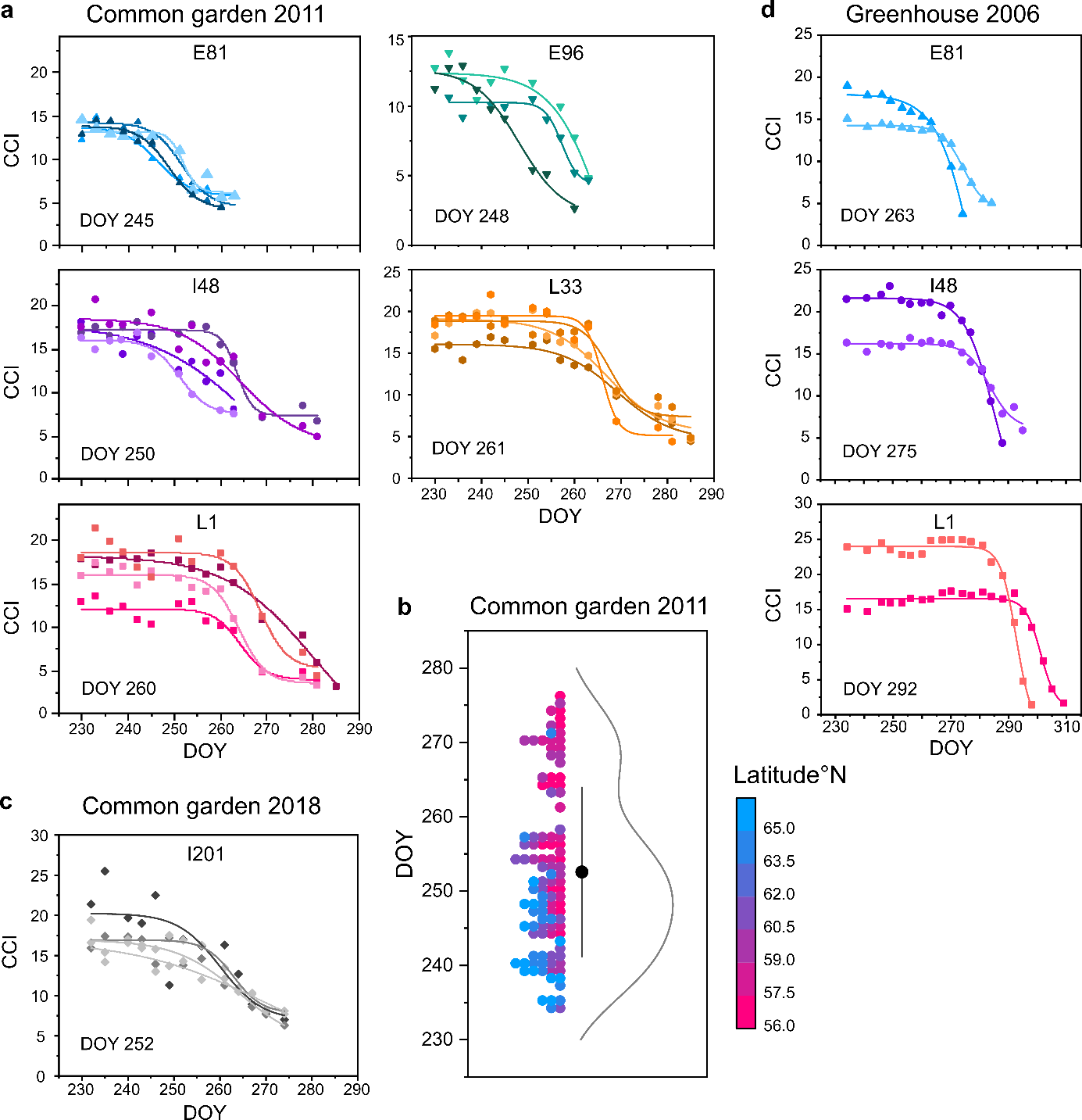


**Fig. S2.** **Autumn senescence onset of aspen genotypes in the field and in the greenhouse.**

Chlorophyll curves and estimated senescence onset date in five Swedish aspen (SwAsp) genotypes selected for this study (**a**) and the distribution of onset date in SwAsp collection in autumn 2011 in the common garden in Sävar, Umeå (**b**). Each data point represents a mean senescence date for a particular genotype (108 genotypes with data from two or more replicate trees) coloured based on their latitude of origin (**b**). The curve represents the Kernel smooth distribution with Scott bandwidth, black points represent the mean date and the whiskers the standard deviation (±SD, **b**). Chlorophyll curves and estimated senescence onset date in four clonal trees of campus tree I201 in autumn 2018 in the common garden (**c**). Senescence onset in the three SwAsp genotypes selected for transcriptome study in the greenhouse in 2006 in Umeå (**d**). The date for senescence onset (**a, c, d**) was estimated based on the curve fitting method of chlorophyll content index (CCI) as described in Lihavainen et al. ^16^. The CCI data for SwAsp genotypes (**a**) and the onset dates for the SwAsp population (**b**) in 2011 were published by Michelson et al. ^15^. The CCI data and experimental setup for the greenhouse experiment in 2006 were published by Francheboud et al. ^13^. The plants were grown in the greenhouse under natural photoperiod and light regime with 15/10 °C day/night temperature. CCI data are mean value of five leaves.


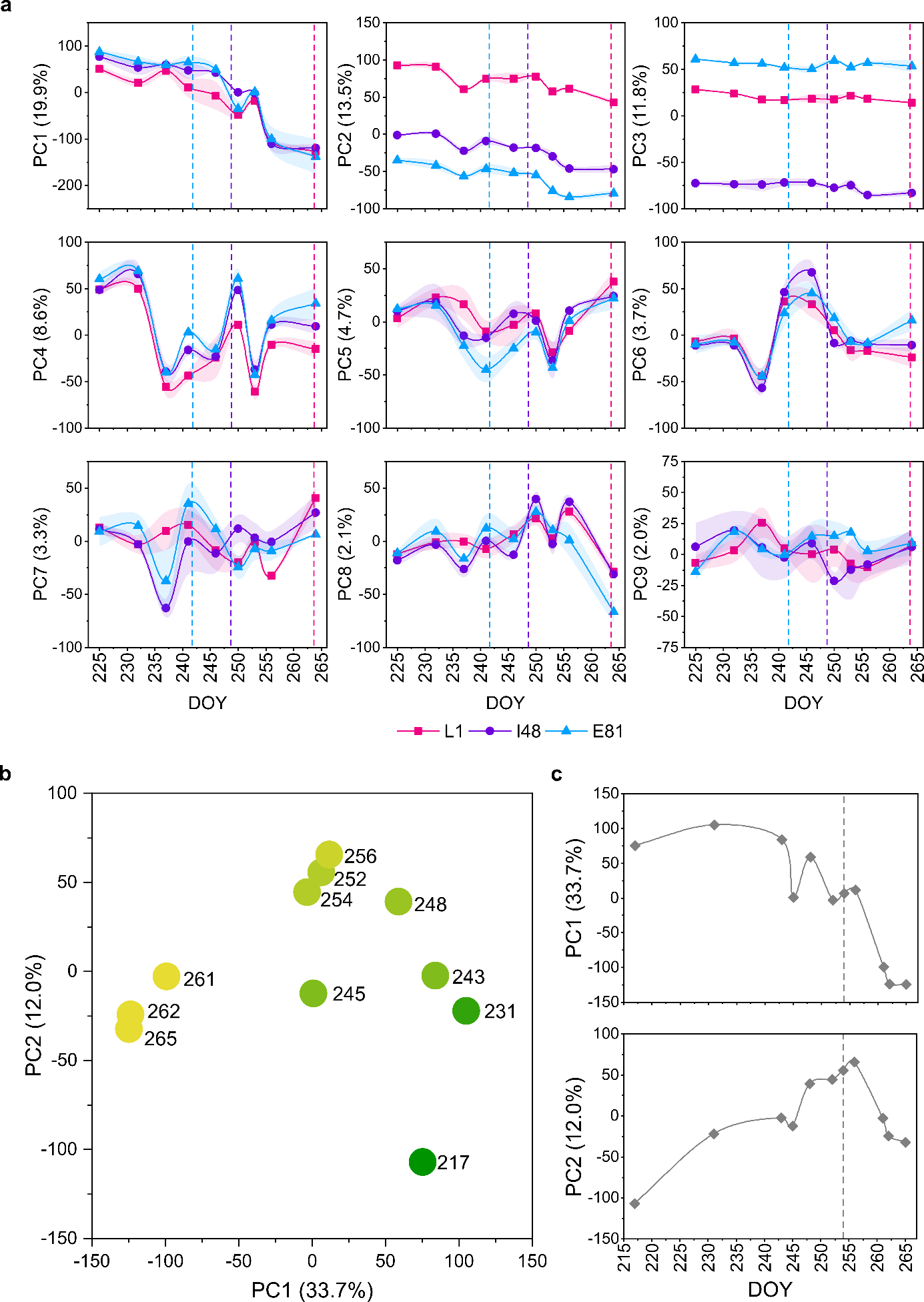


**Fig. S3.** **Principal component analysis (PCA) depicting the global transcriptome profile during autumn.**

Time-dependent patterns of principal components (PC) of transcriptome data in three SwAsp genotypes in autumn 2018 (**a**). Data are mean PC scores ± SE, n=2-3 in each time point per SwAsp genotype, see the details for n in a particular genotype and time point in Source Data file. Scores plot (**b**) and time-dependent patterns of PC (**c**) in aspen genotype I201 in autumn 2011. Data are PC score of one tree, n=1. The day of the year (DOY) is marked in the scores plot (**b**). Dashed vertical lines represent the estimated senescence onset date in a particular genotype. Source data are provided as Source Data files.


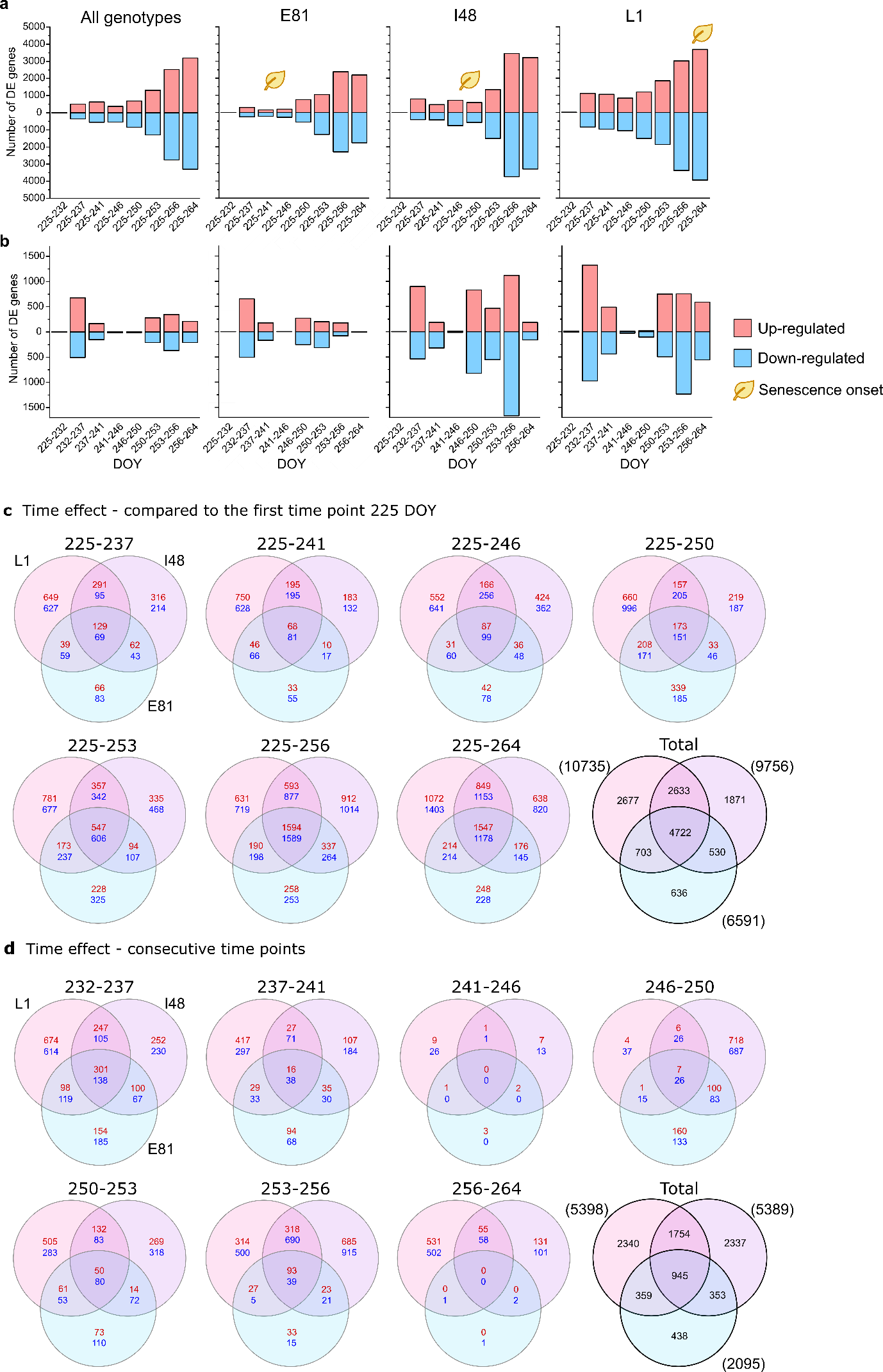


**Fig. S4.** **Differentially expressed genes in three SwAsp genotypes in autumn 2018.**

The effect of time on gene expression were tested with DESeq2 (Wald test) in two different ways: by comparing the expression to the first time point 225 DOY (**a**) and between consecutive time points (**b**) considering all genotypes and within each individual genotype. Genes with FDR adjusted *P*-value <0.01 (two-sided) were considered significantly differentially expressed and the results were filtered based on log_2_ fold change cut-off 0.5. Venn diagrams were constructed to identify similarly affected genes and those that displayed unique responses across time points between SwAsp genotypes (**c, d**). Yellow leaf marks the estimated start of senescence in each genotype. Source data are provided as Source Data files.


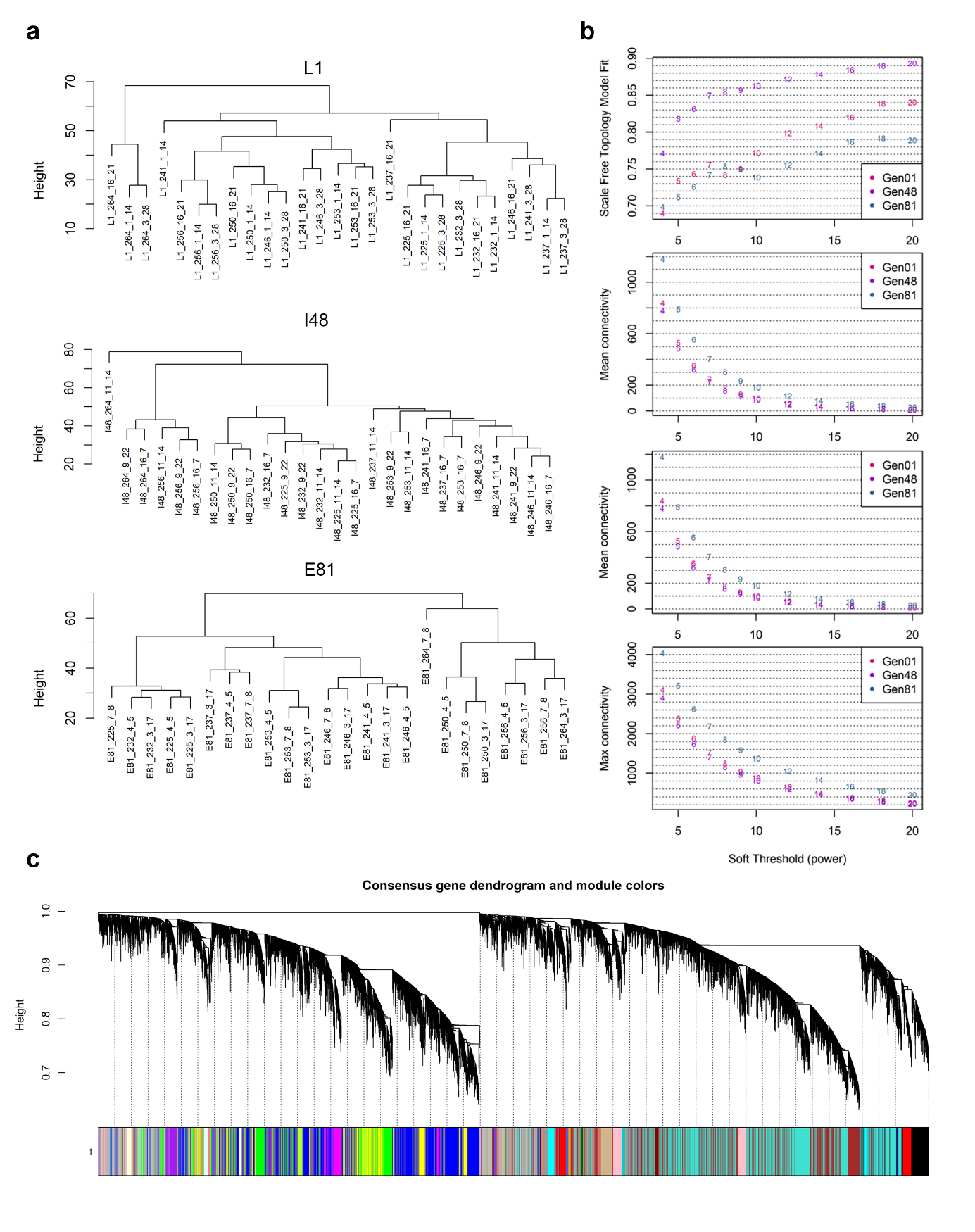


**Fig. S5.** **Weighted gene co-expression network analysis (WGCNA) statistics of 2018 data.**

Sample clustering (**a**), connectivity statistics (**b**) and consensus dendrogram with module colours (**c**).


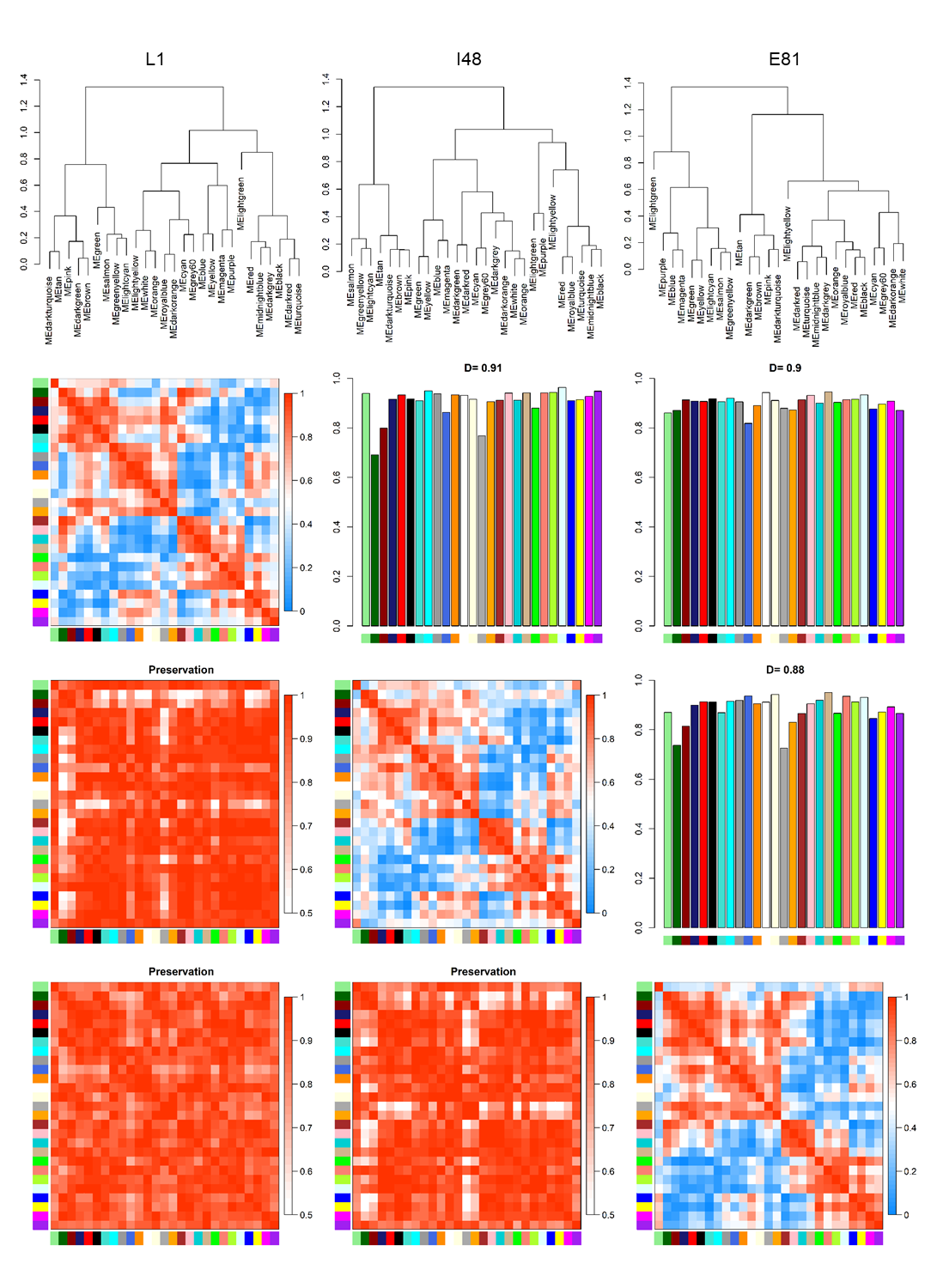


**Fig. S6.** **Preservation of WGCNA modules and module relationships in three SwAsp genotypes in autumn 2018.**

See details of the analysis in Supplementary Methods.


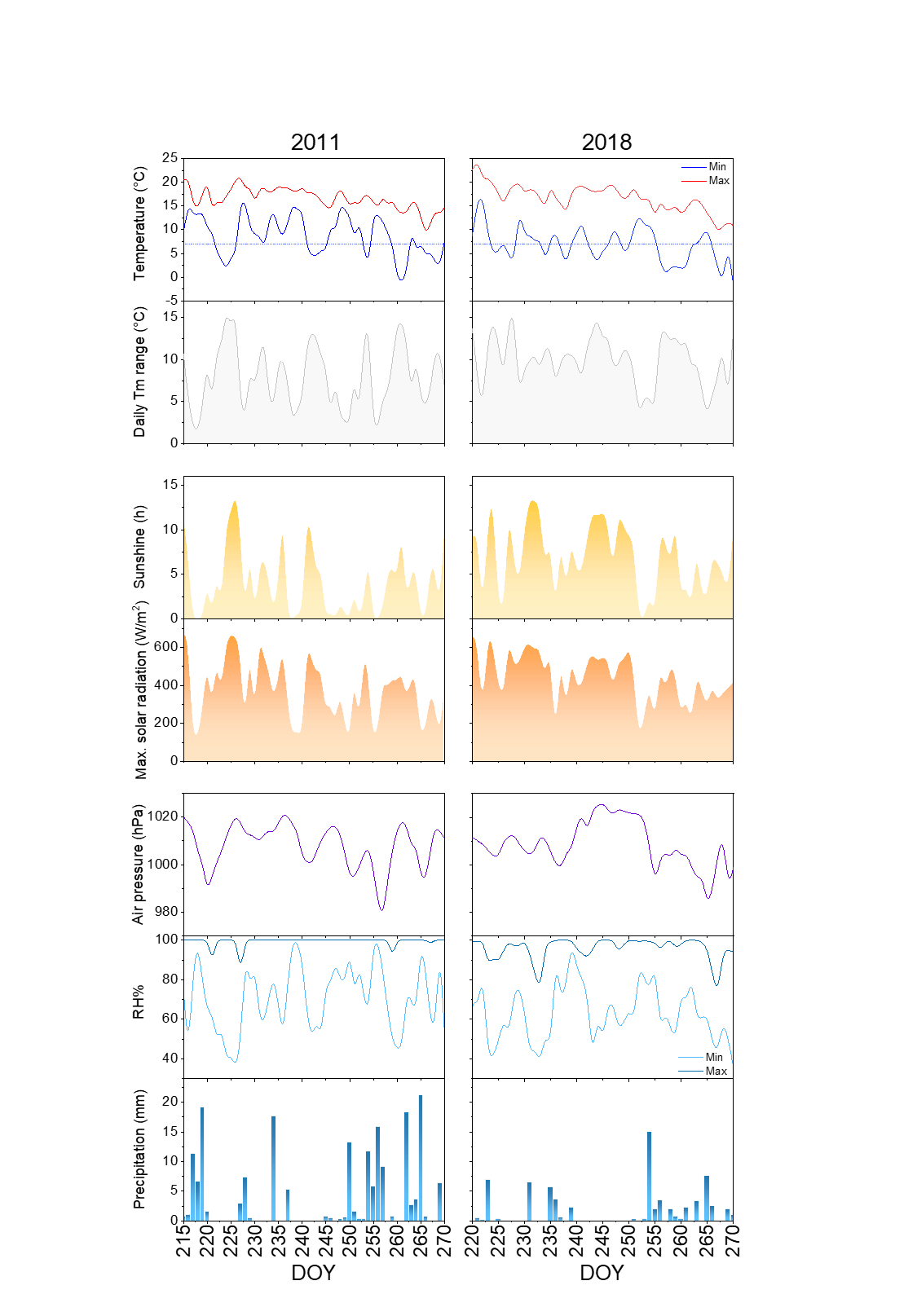


**Fig. S7.** **Weather parameters in autumn 2011 and 2018 in Umeå, Sweden.**

Air temperature (°C), precipitation (mm), air pressure (kPa), relative humidity (RH%), daily sunshine hours (global radiation over 120 kW/m^2^) and maximum solar radiation (global radiation W/m^2^) data were obtained for Umeå region from Swedish Hydrological and Meteorological Institute (SHMI, [www.shmi.se](http://www.shmi.se)) and from the TFE weather station located at the Umeå University campus ((<http://www8.tfe.umu.se/TFE-vader/>, Department of Applied Physics and Electronics, Umeå University). Air temperature below 7 °C was considered as chilling temperature (blue dashed line).

**Table S1.** Sunshine, precipitation and mean air temperature in July-October in 2011 and 2018 and over ten years (2010-2020) in Umeå. Values in red are above and values in blue are below the 10-year average.

|  |  | **2011** | **2018** | **2010-2020** | | | |
| --- | --- | --- | --- | --- | --- | --- | --- |
|  | **Month** |  |  | **Mean** | **SD** | **Min** | **Max** |
| **Sum of sunshine hours** | Jul | 304.1 | 394.5 | 349.3 | 64.0 | 225.1 | 394.5 |
|  | Aug | 178.7 | 248.4 | 213.6 | 49.3 | 172.0 | 291.1 |
|  | Sep | 94.3 | 185.6 | 139.9 | 64.6 | 92.0 | 218.0 |
|  | Oct | 137.8 | 126.8 | 132.3 | 7.8 | 48.4 | 161.7 |
| **Days of precipitation** | Jul | 9 | 5 | 7 | 3 | 2 | 19 |
|  | Aug | 11 | 10 | 11 | 1 | 4 | 19 |
|  | Sep | 20 | 16 | 18 | 3 | 5 | 20 |
|  | Oct | 11 | 12 | 12 | 1 | 6 | 21 |
| **Sum of precipitation (mm)** | Jul | 63.0 | 68.2 | 47.1 | 35.4 | 4.5 | 139.9 |
|  | Aug | 74.3 | 28.6 | 63.5 | 35.4 | 18.2 | 123.9 |
|  | Sep | 116.6 | 44.1 | 67.5 | 27.1 | 11.2 | 116.6 |
|  | Oct | 59.2 | 54.3 | 69.1 | 51.6 | 6.2 | 166.4 |
| **Mean air temperature** | Jul | 17.0 | 19.2 | 18.1 | 1.6 | 14.2 | 19.2 |
|  | Aug | 14.5 | 15.1 | 14.8 | 0.4 | 13.3 | 15.2 |
|  | Sep | 11.7 | 10.1 | 10.9 | 1.1 | 8.7 | 11.7 |
|  | Oct | 4.9 | 3.3 | 4.1 | 1.1 | 2.4 | 5.7 |


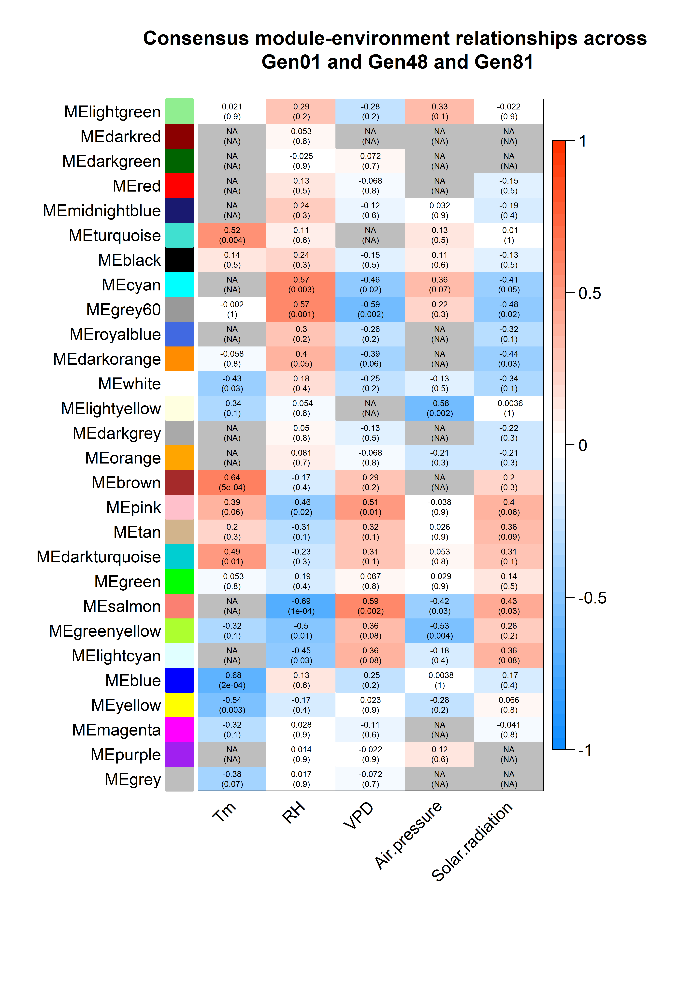

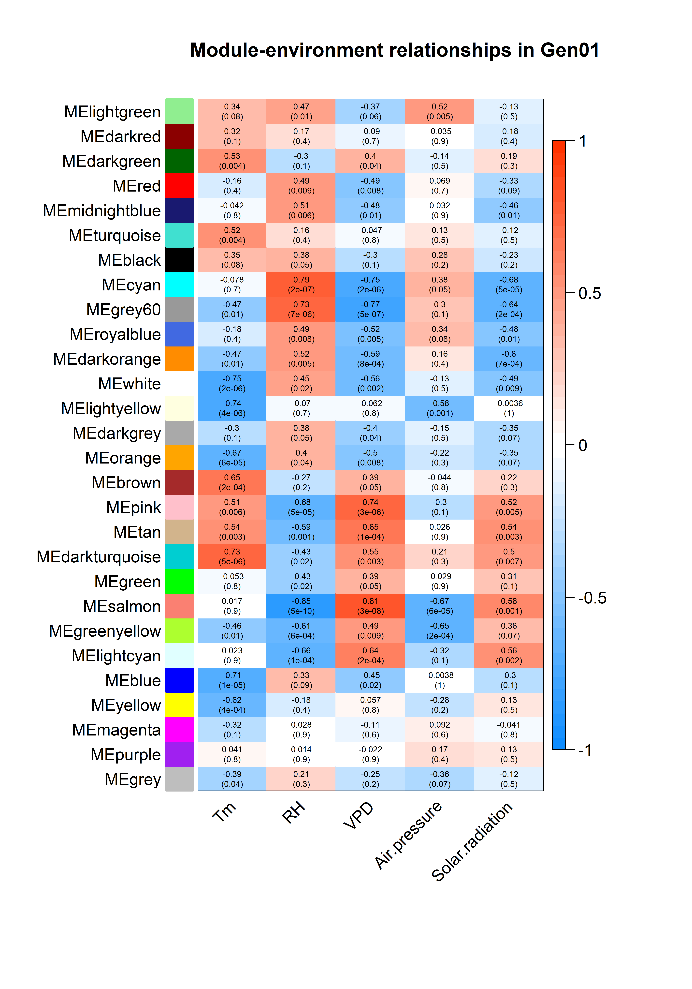


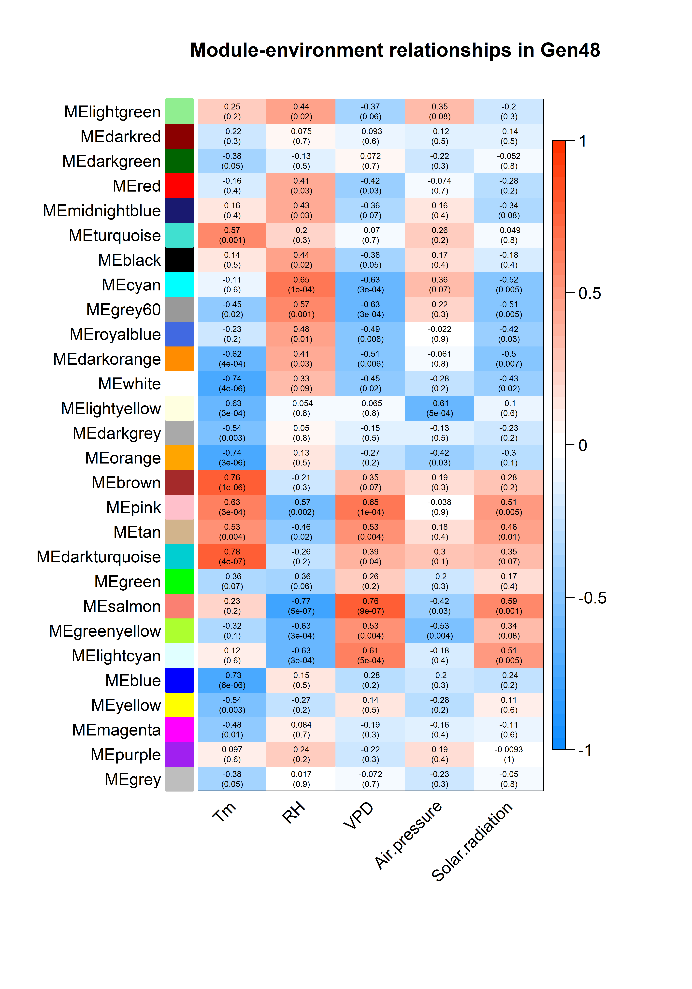

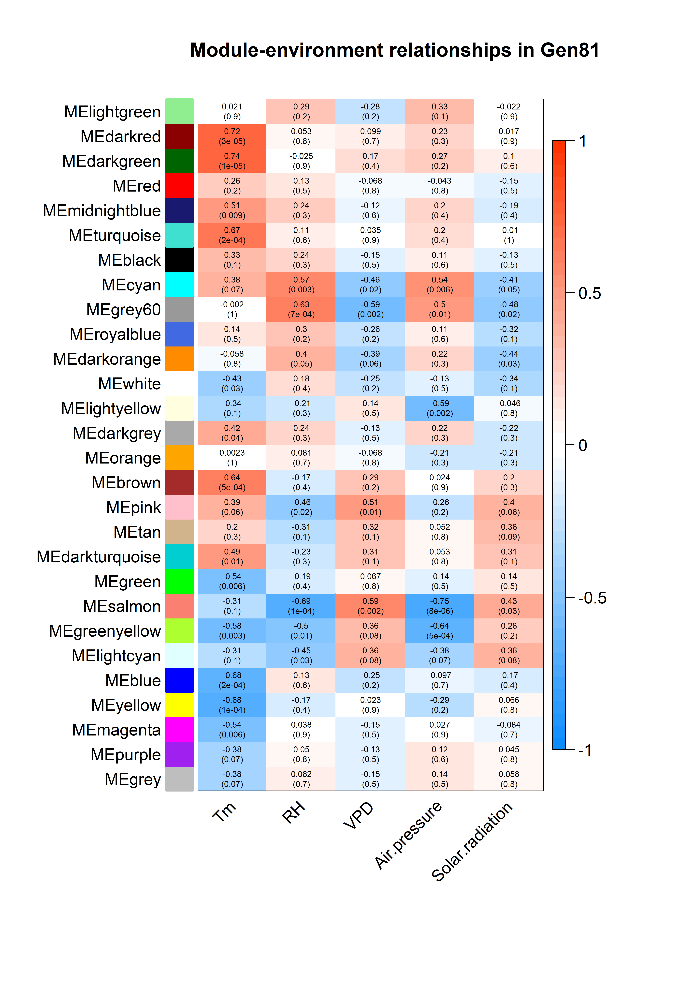


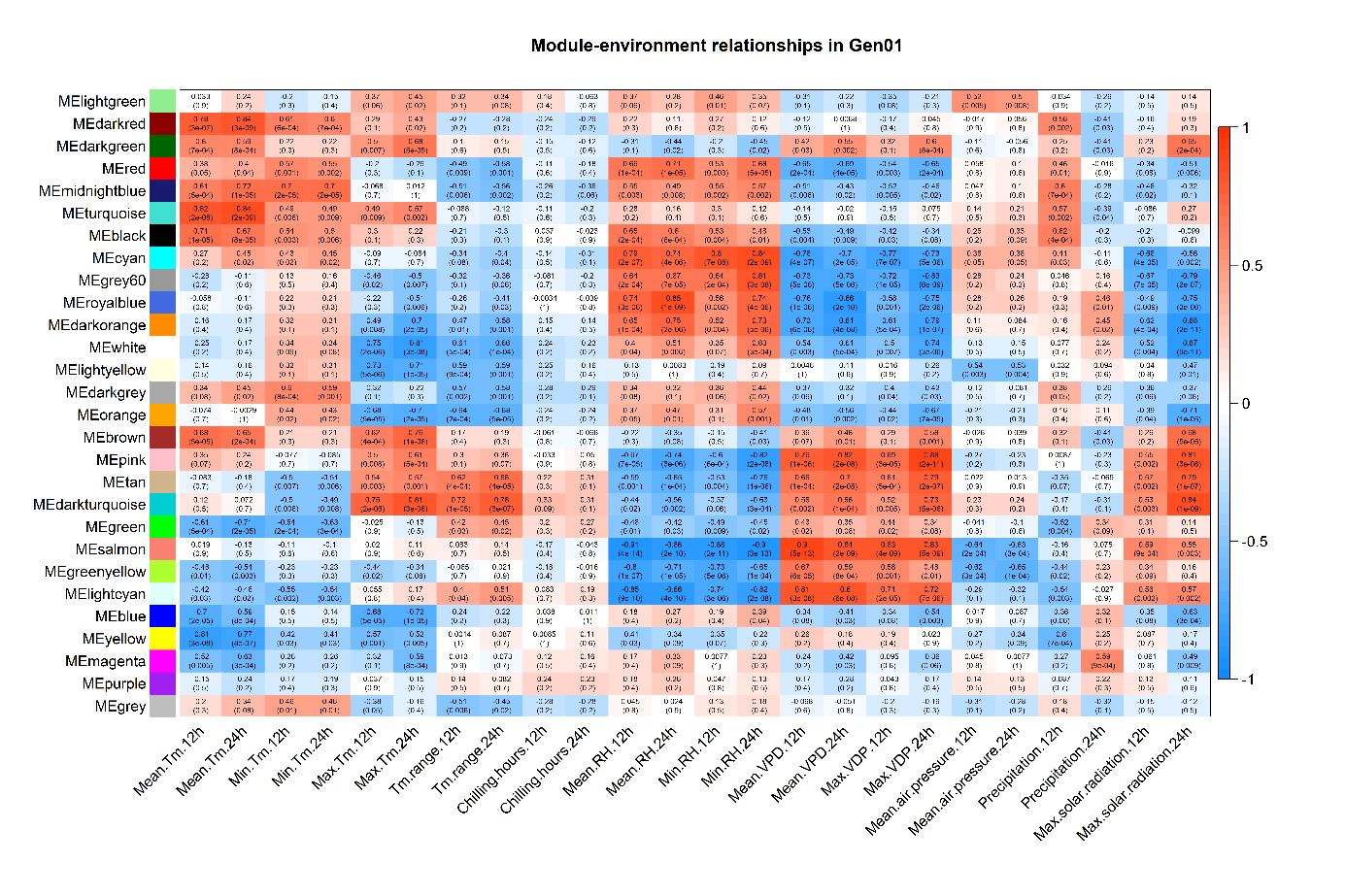


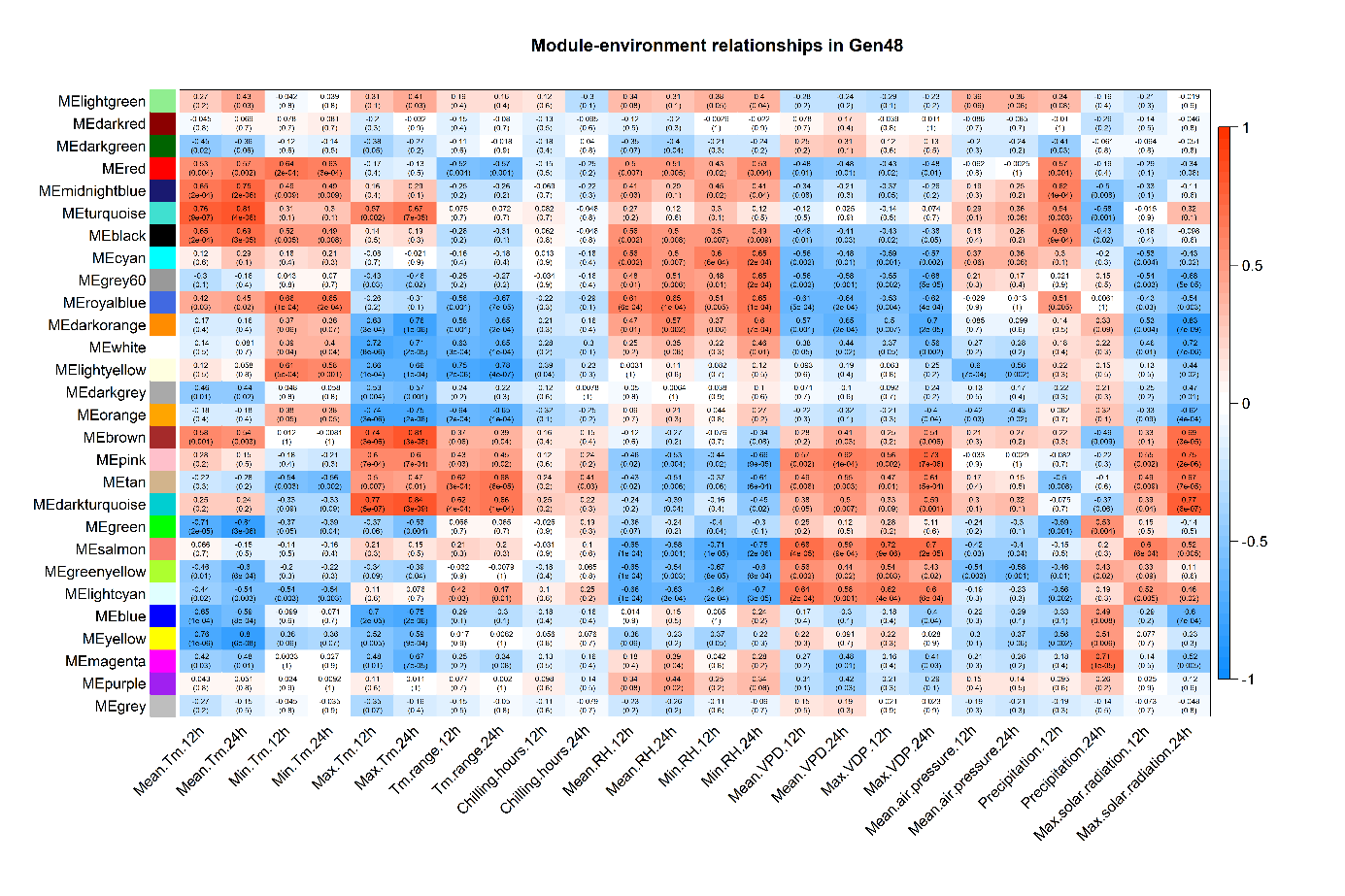


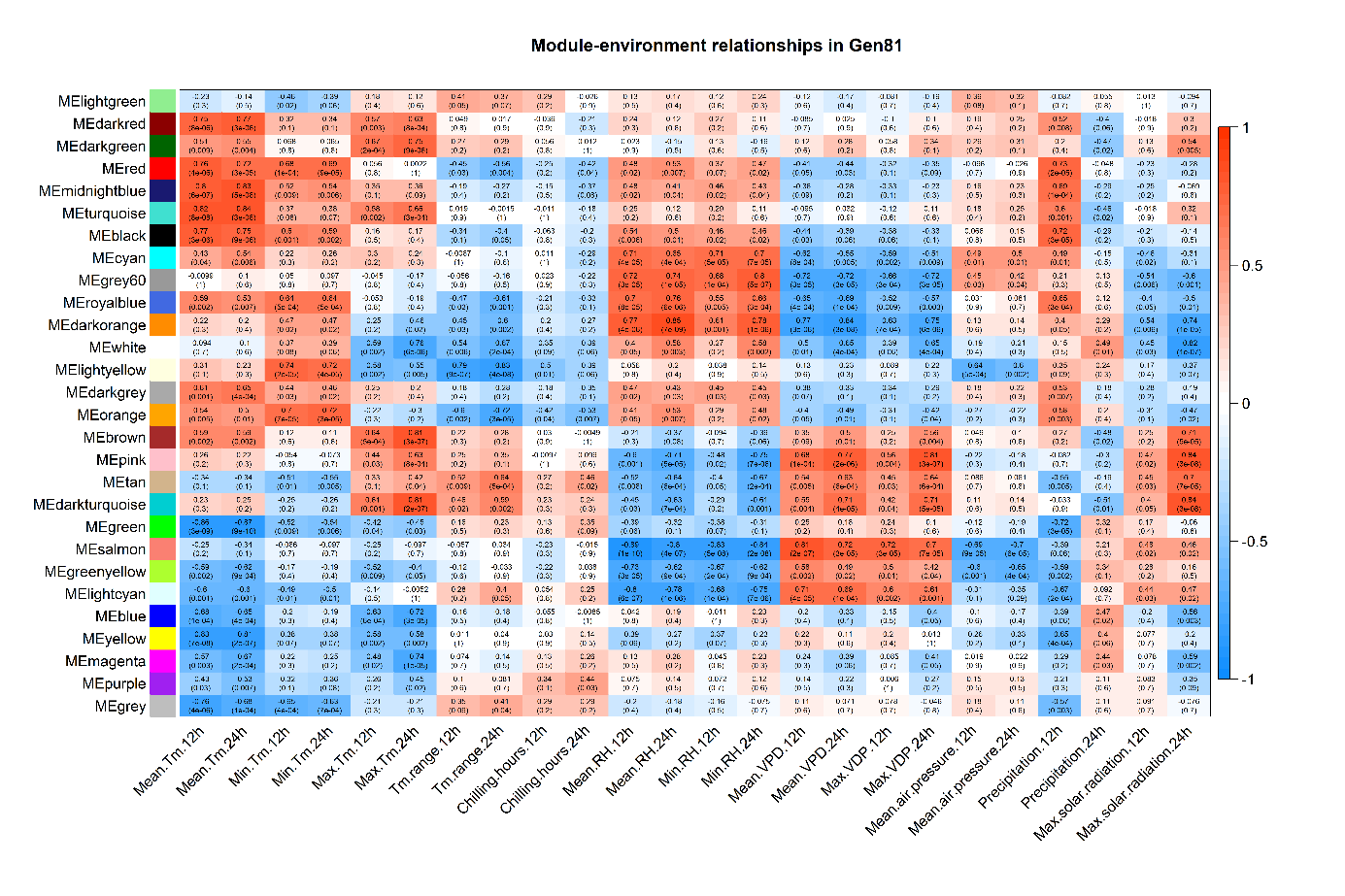


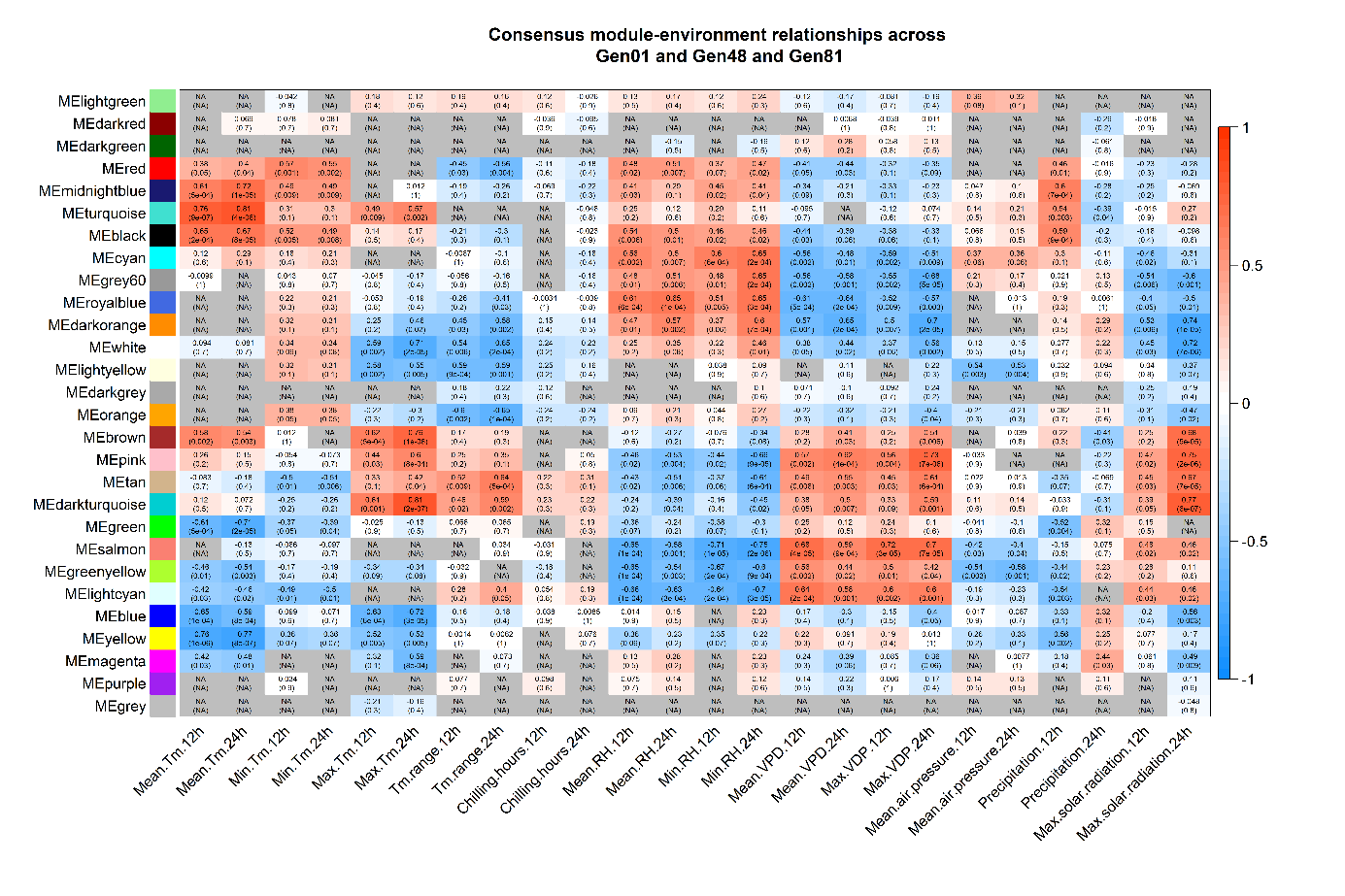


**Fig. S8.** **Consensus and genotypic (SwAsp) correlation plots of signature expression of WGCNA eigengenes with weather parameters in autumn 2018.**

Correlations were performed with weather parameters at the time of sampling and with parameters over the past 12 and 24 hours. Cells are coloured based on Pearson correlation coefficient *r*. Correlation was considered significant with *P*<0.05 (two-sided).


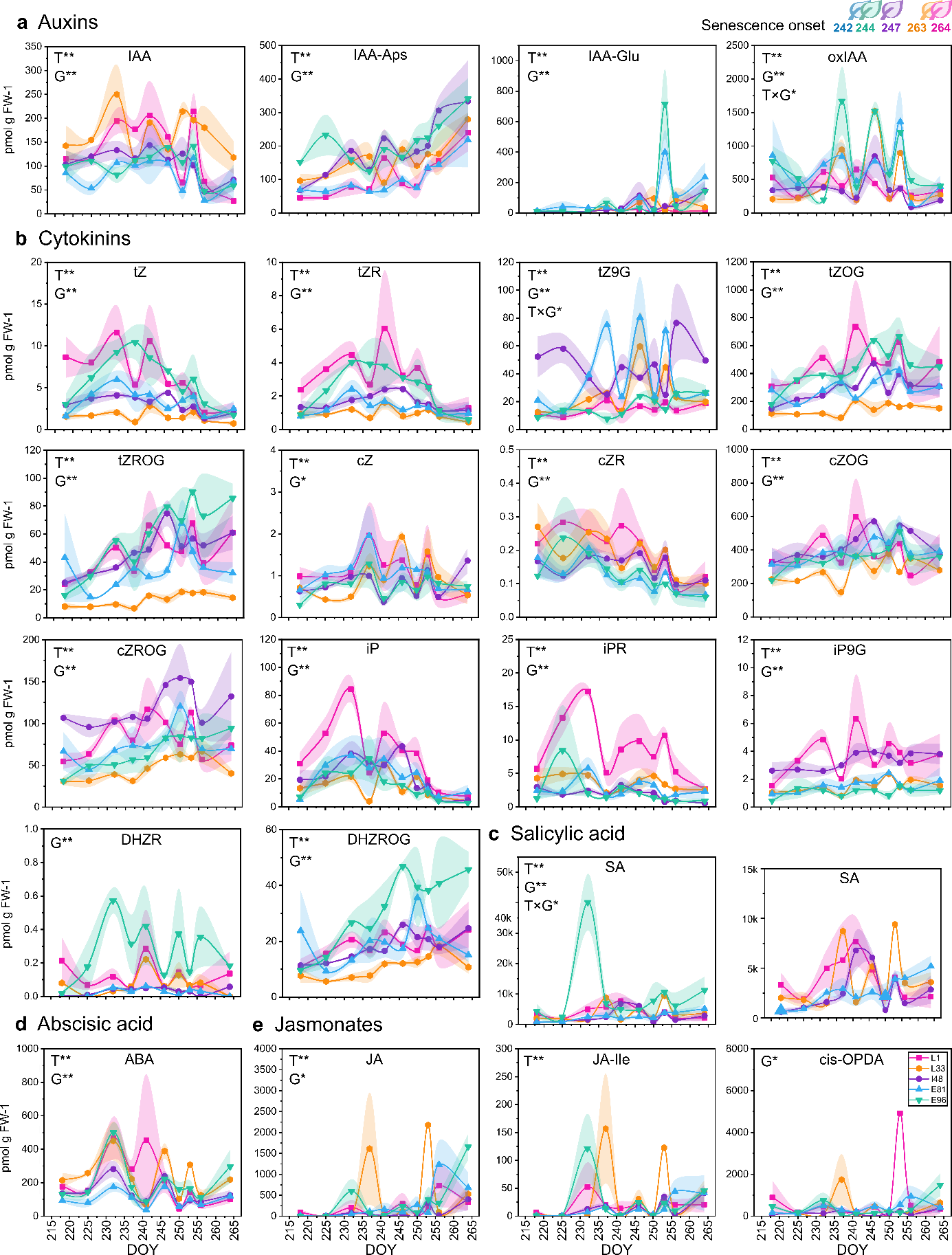


**Fig. S9. Levels of auxin (a), cytokinin (b), salicylic acid (c), abscisic acid (d), jasmonic acid (e) and their metabolites in the leaves of five SwAsp genotypes in autumn 2018.**

The data are mean ± SE (highlighted area), n=2-3 in each time point per SwAsp genotype, except n=1-3 in jasmonates. The effects of time (T), genotype (G) and their interaction (T×G) were tested with two-way ANOVA (FDR adjusted *P*-value <0.05* <0.01**). The details of phytohormone analysis and statistical results are in Supplementary Data 14. Source data are provided as Source Data files.


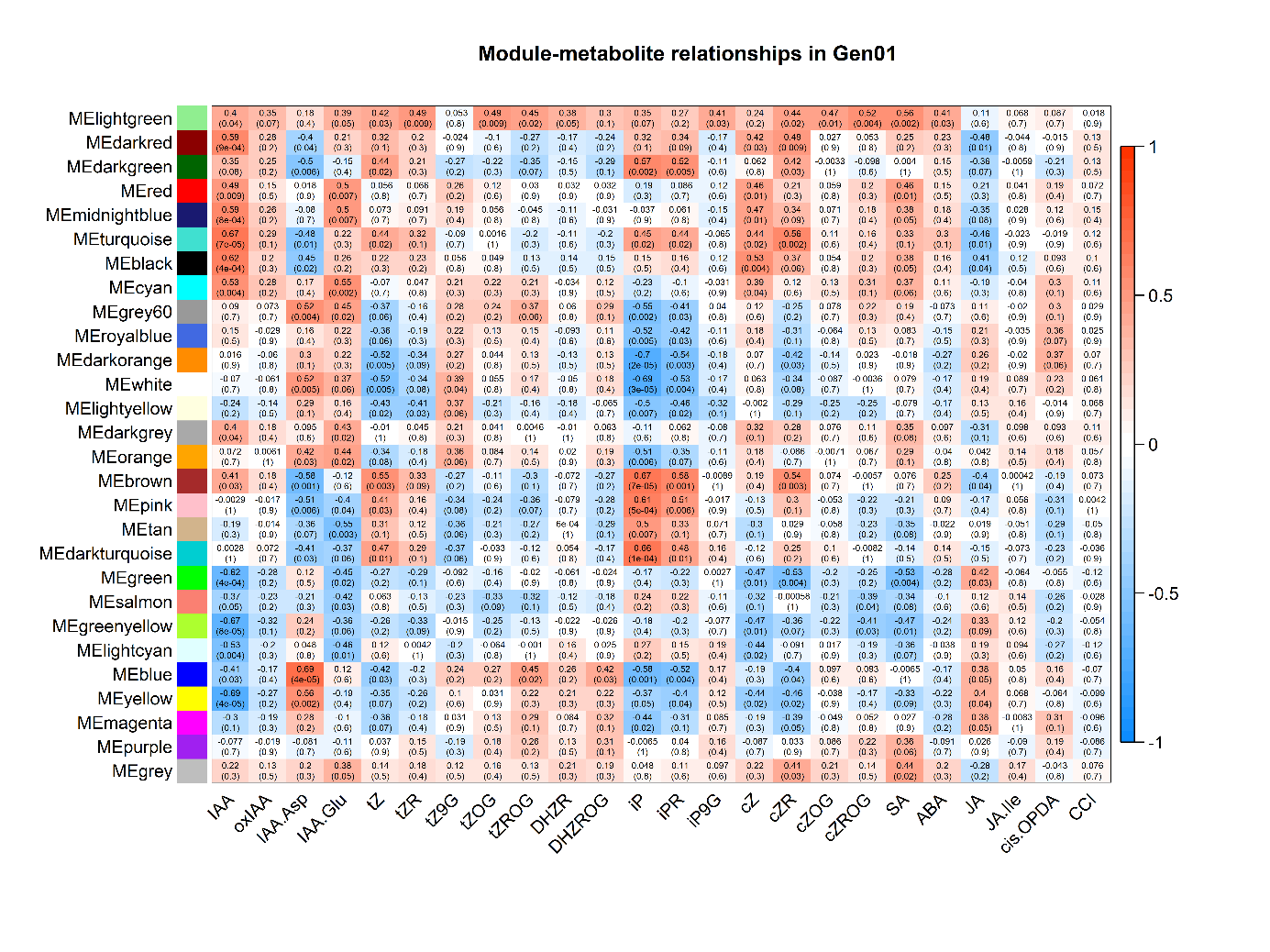


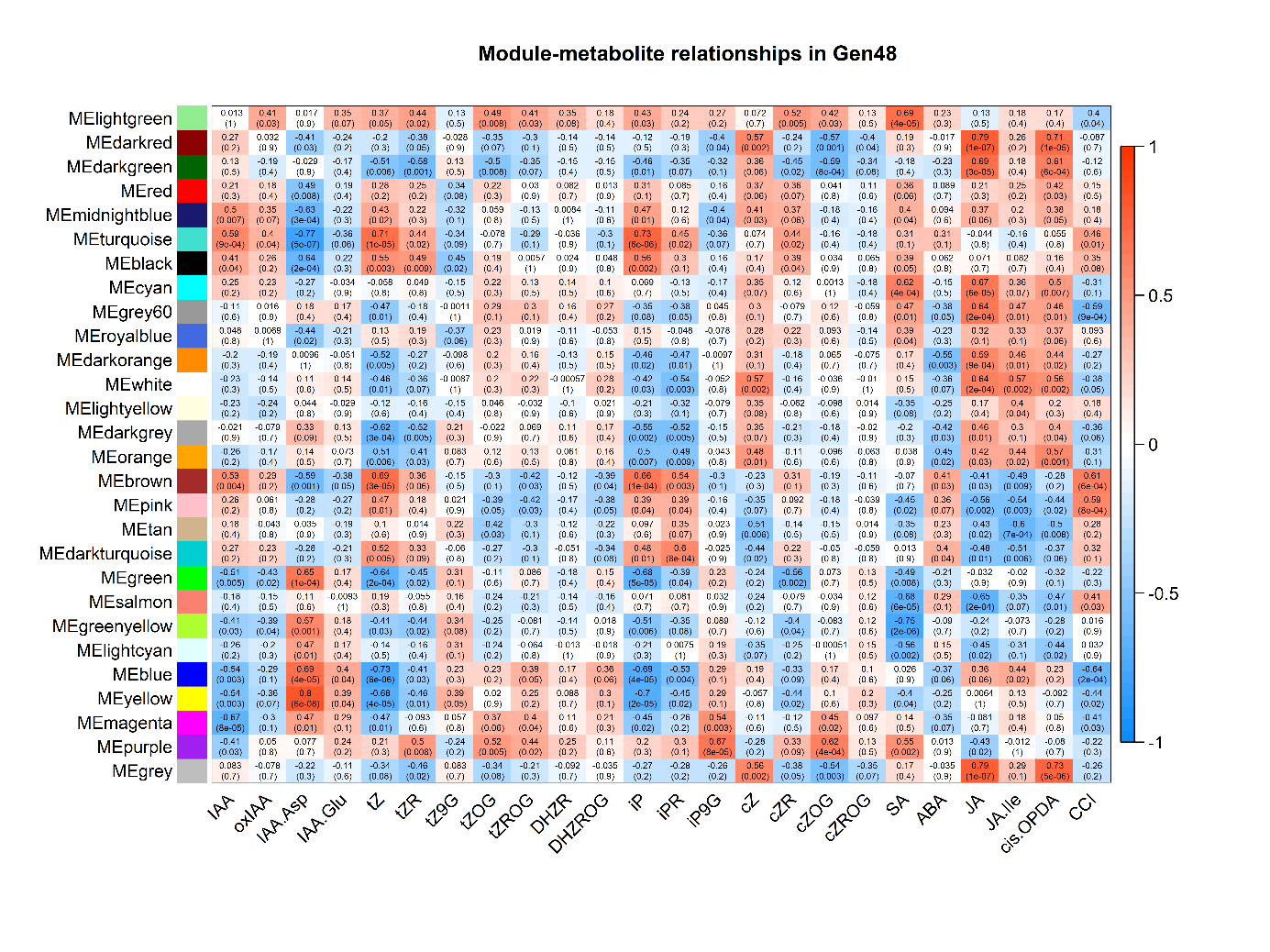


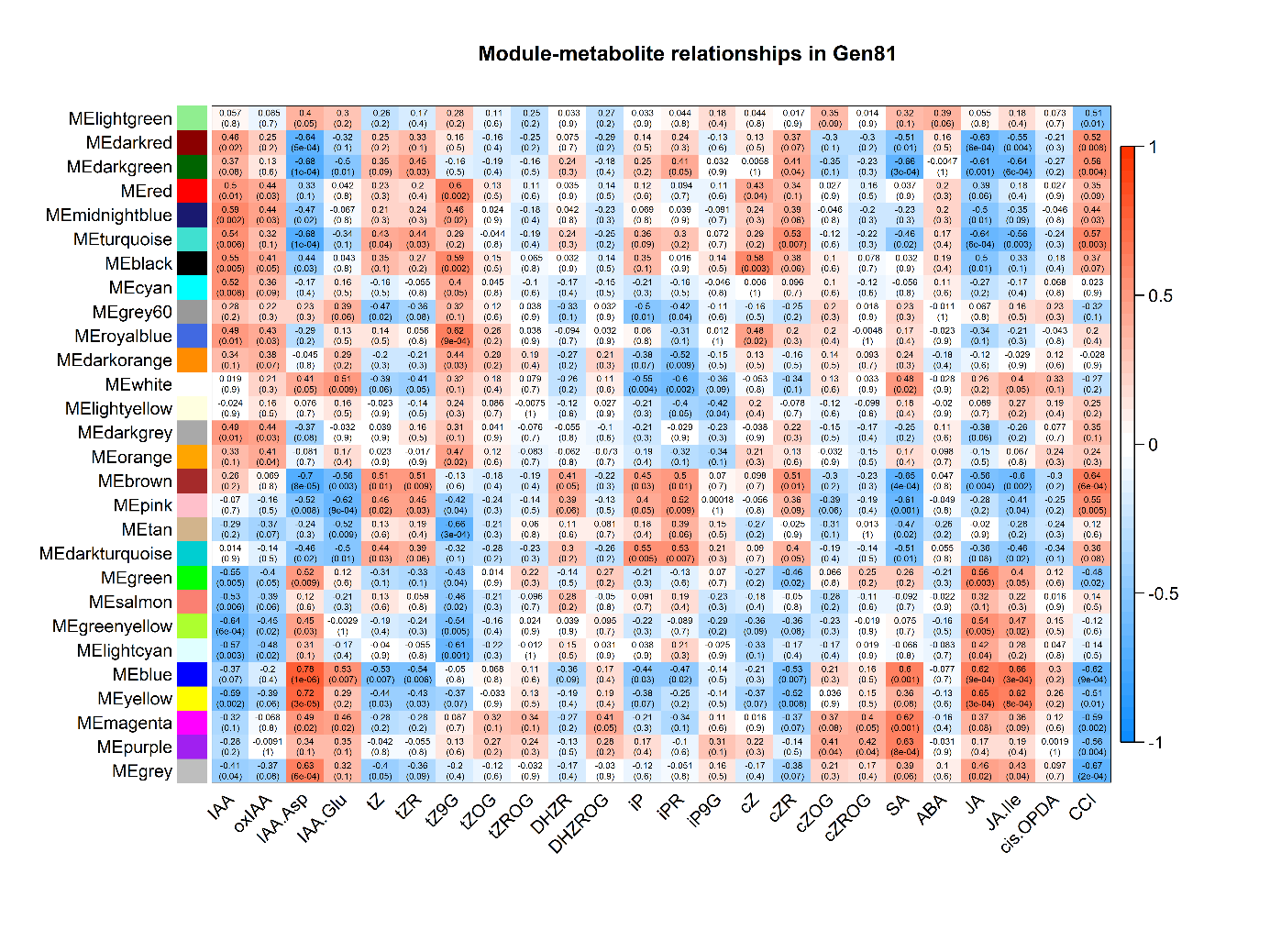


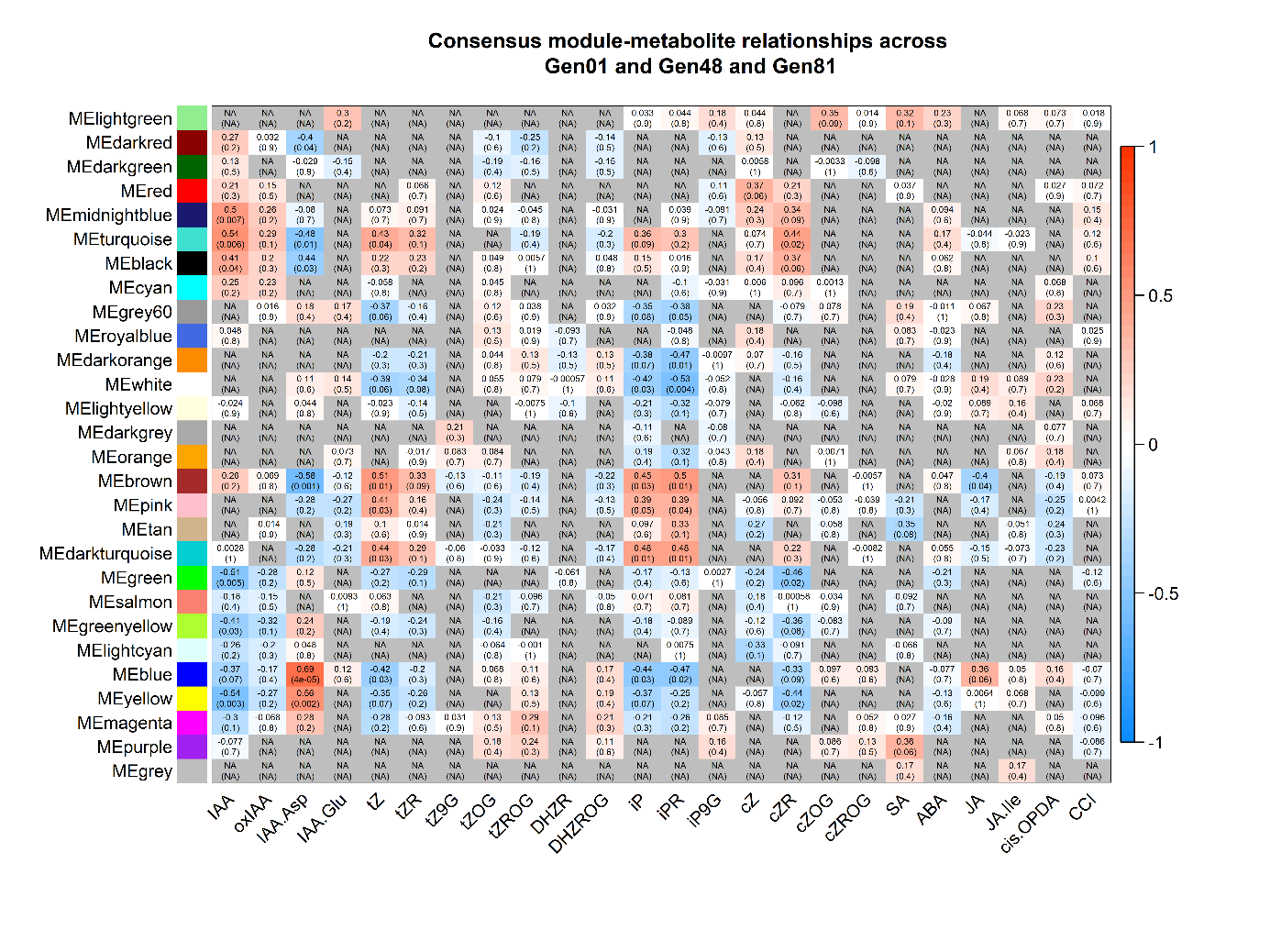


**Fig. S10. Consensus and** **genotypic (SwAsp) correlation plots of signature expression of WGCNA eigengenes with metabolic markers: phytohormone levels (pmol/g FW) and chlorophyll content index (CCI) in 2018.**

Cells are coloured based on Pearson correlation coefficient *r*. Correlation was considered significant with *P*<0.05 (two-sided).


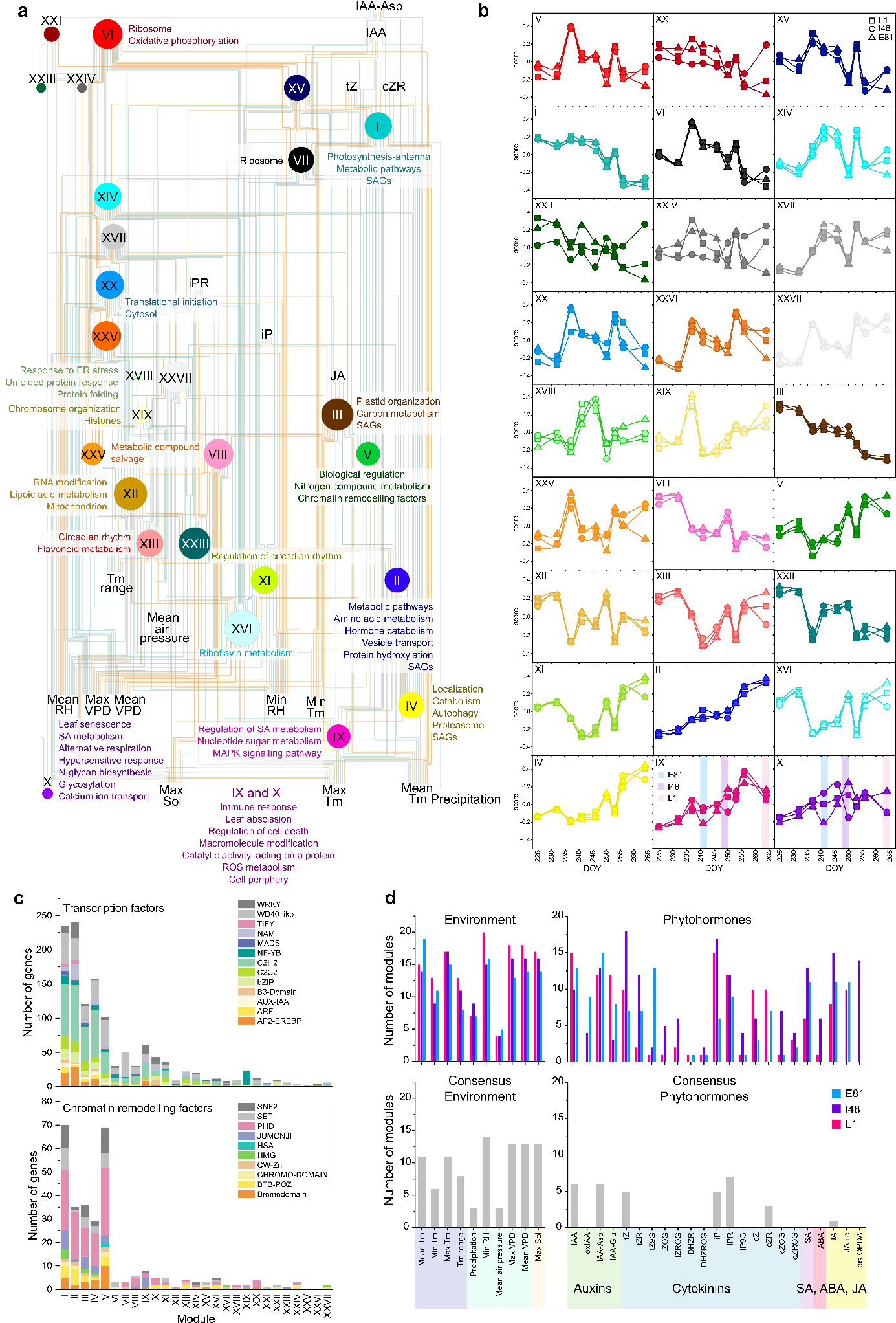


**Fig. S11. Hierarchical correlation network of eigengenes, weather parameters (past 24 hours) and phytohormone levels in three SwAsp genotypes in autumn 2018 (a).**

Nodes are coloured based on the eigengenes, their size is proportional to their degree (connectivity) and edges are coloured based on positive (orange) and negative (blue) correlation (Pearson *r*, **a**). Only the edges with *P*-value <0.05 (two-sided) in all three genotypic networks are shown (**a**). The signature expression patterns of WGCNA eigengenes (**b**). The values are mean scores of the first principal component of the module (n=2-3 in each time point per genotype). The abundance of transcription factor families and chromatin remodelling factors in each gene module (**c**). The number of eigengenes (module patterns) with significant correlation with environmental factors and phytohormone levels in consensus data and in each SwAsp genotype (**d**). Eigengene scores of the replicate samples and the results of GO term and KEGG pathway enrichment analyses are in Supplementary Data 4. Modules are numbered based on the descending number of assigned genes. Source data are provided as Source Data files.

**
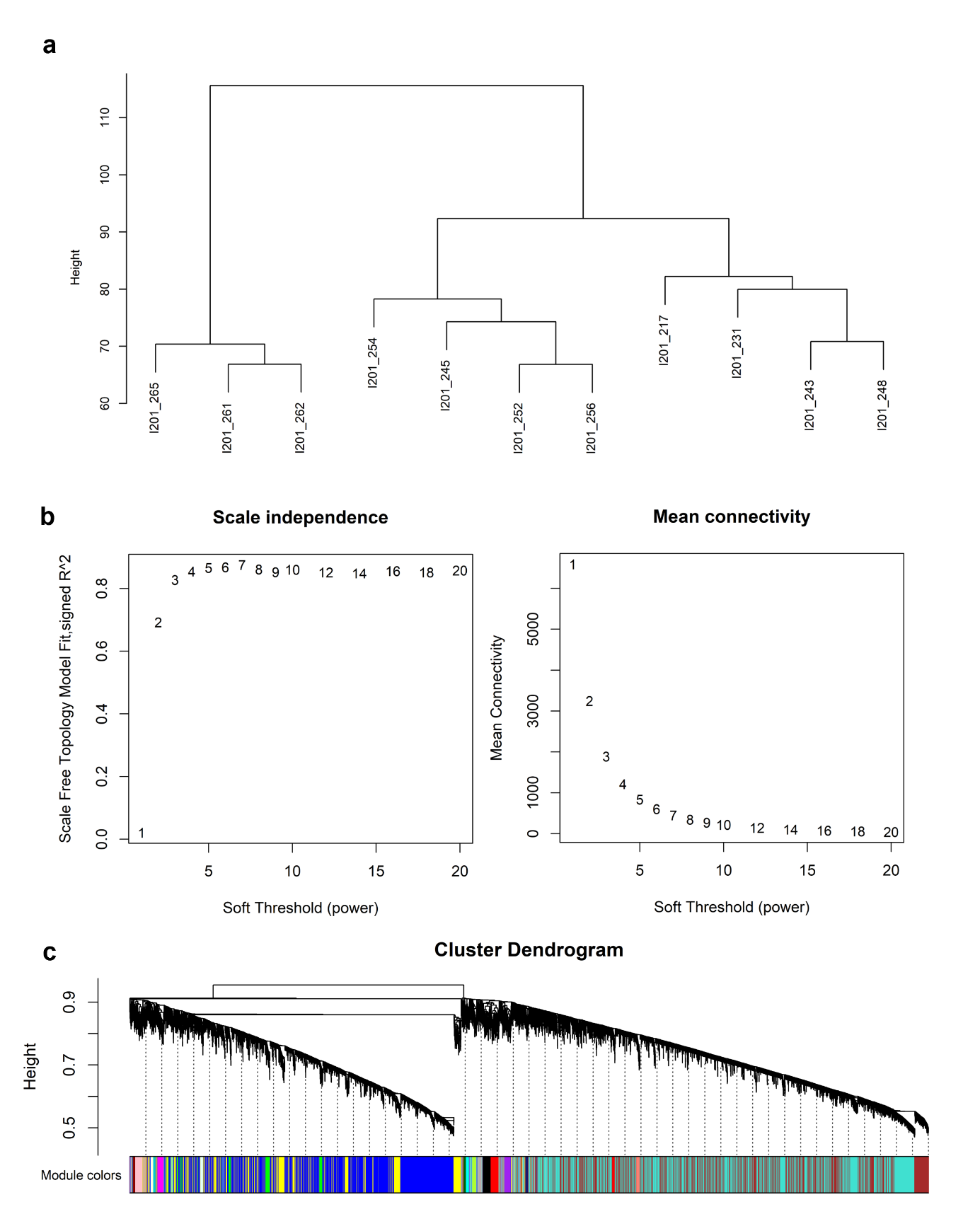
**

**Fig. S12.** **Weighted gene co-expression network analysis (WGCNA) statistics of the 2011 data.**

Sample clustering (**a**) connectivity statistics (**b**) and dendrogram with module colours (**c**) (note that the modules are different than in the 2018 WGCNA data).

**
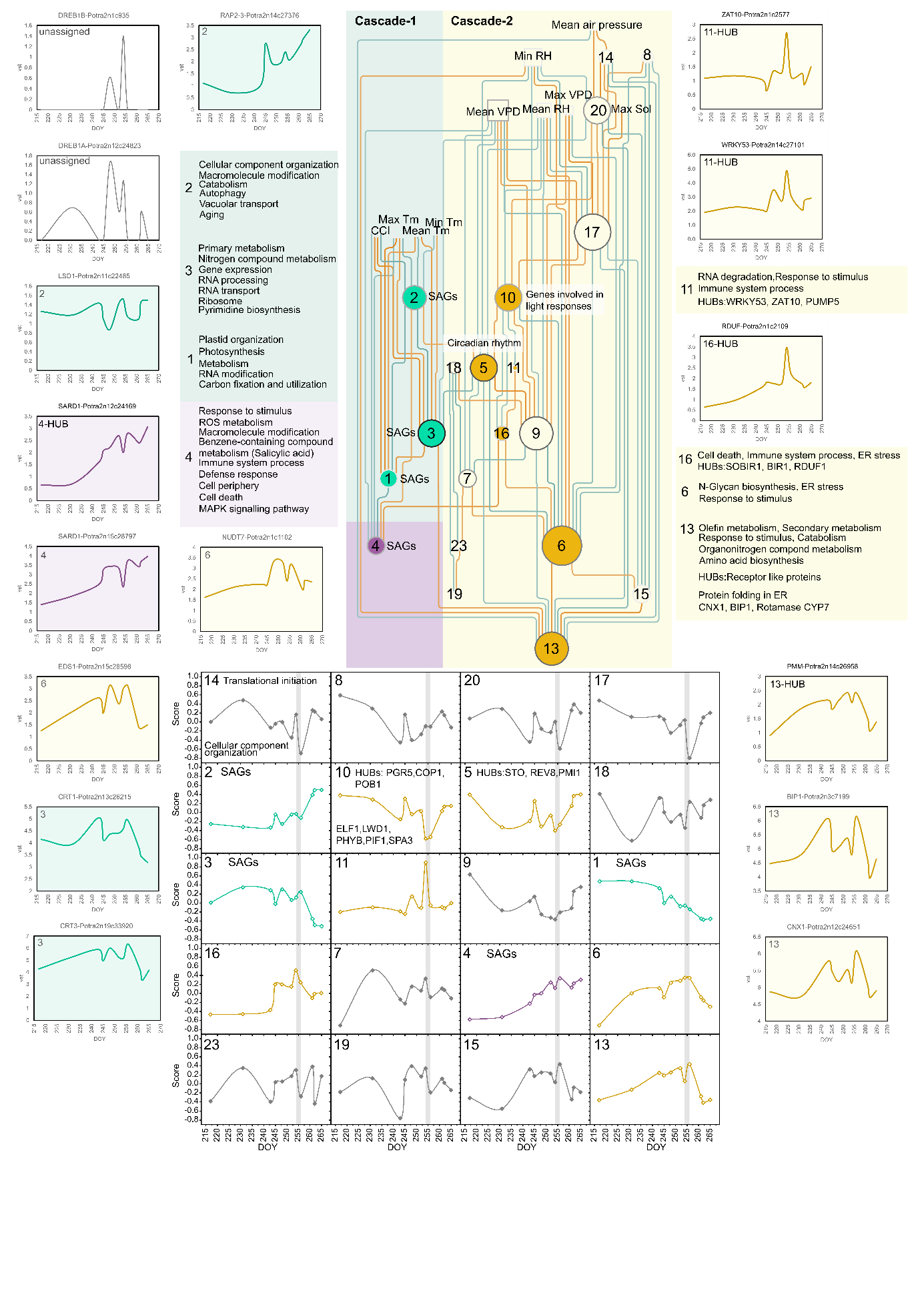
**

**Fig. S13.** **Weighted gene co-expression network analysis (WGCNA) eigengene patterns and the expression of genes in selected modules in I201 in autumn 2011.**

The network structure displayed broadly two cascades: 1) modules generally up- or down-regulated (including senescence-associated genes-SAGs) 2) modules showing short-term variations during autumn. A module enriched with SAGs, salicylic acid (SA) biosynthesis and innate immunity related genes were co-expressed with modules in both cascades (4). The expression of module hub *SARD1* was in general enhanced during autumn, but transiently repressed at senescence onset in I201 in autumn 2011 like in other SwAsp genotypes in autumn 2018. SA-mediated module (4) showed a positive correlation with cell death regulation (16, genes encoding receptors as hubs) upstream of modules enriched with genes involved in glycosylation (6), endoplasmic reticulum (ER) stress responses (6, 13), metabolic processes (13) along with genes encoding receptor like proteins (hubs in module 13). The shaded vertical line represents senescence onset date in genotype I201 in autumn 2011. Details of Gene Ontology (GO) and Kyoto Encyclopaedia of Genes and Genomes (KEGG) pathway enrichment analyses and the list of top hub genes in the modules are in Supplementary Data 6. Eigengene patterns are scores of the first principal component (PC1) of the module and the gene expression patterns are VST(variance stabilizing transformation)-normalised values, n=1 in each time point. The modules are numbered based the descending number of assigned genes (note that the modules are different than in the 2018 WGCNA data). Nodes are coloured based the assigned cascade, their size is proportional to their degree (connectivity) and edges are coloured based on positive (orange) and negative (blue) correlation (Pearson *r* with *P*-value <0.05 two-sided). Source data are provided as Source Data files.


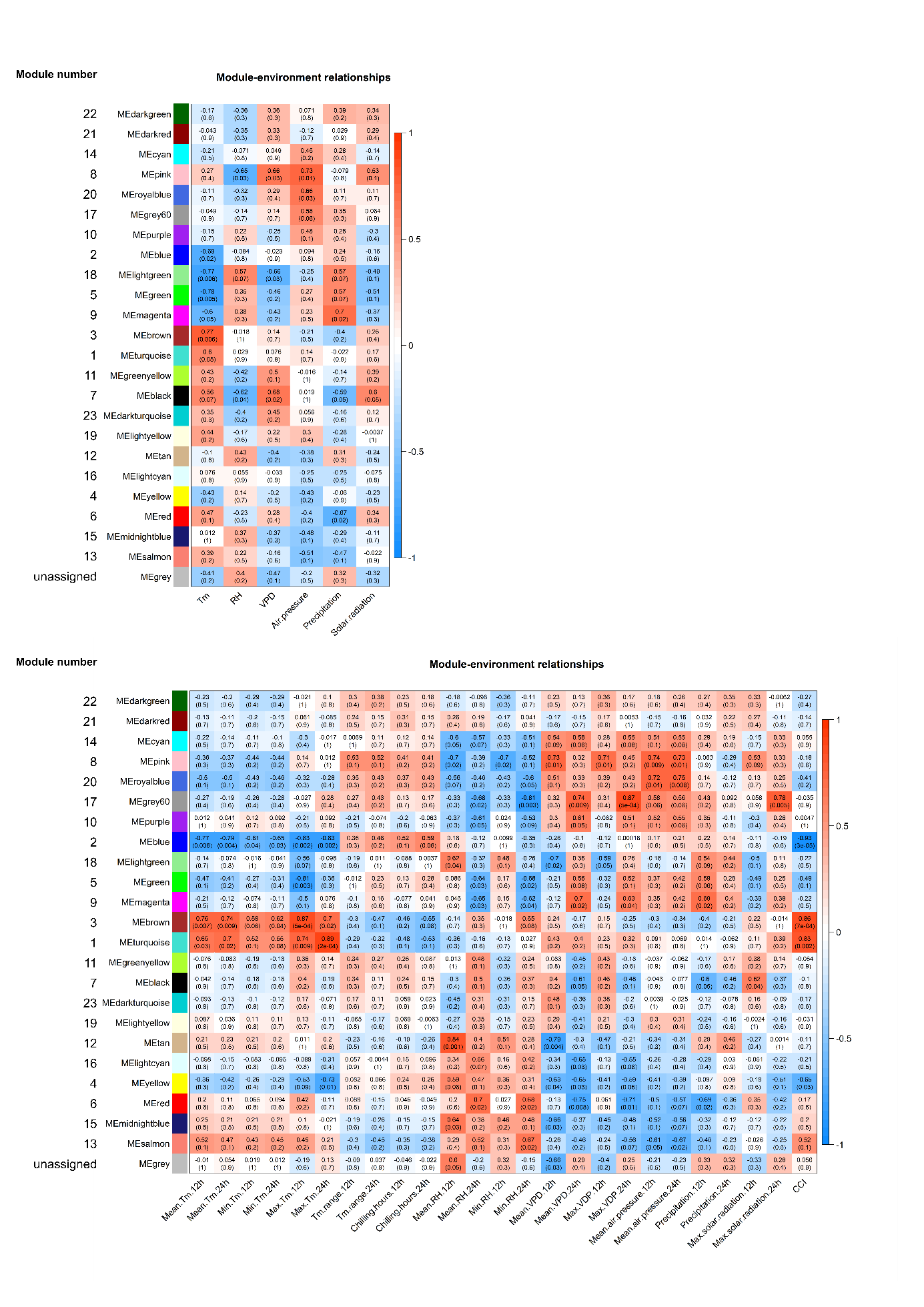


**Fig. S14.** **Correlation plot of signature expression of WGCNA eigengenes with weather parameters and chlorophyll content index (CCI) in genotype I201 in autumn 2011.** Correlations were performed with the weather parameters at the time of sampling and with parameters over the past 12 and 24 hours. Cells are coloured based on Pearson correlation coefficient *r* (note that the modules are different than in the 2018 WGCNA data). Correlation was considered significant with *P*<0.05 (two-sided).

*
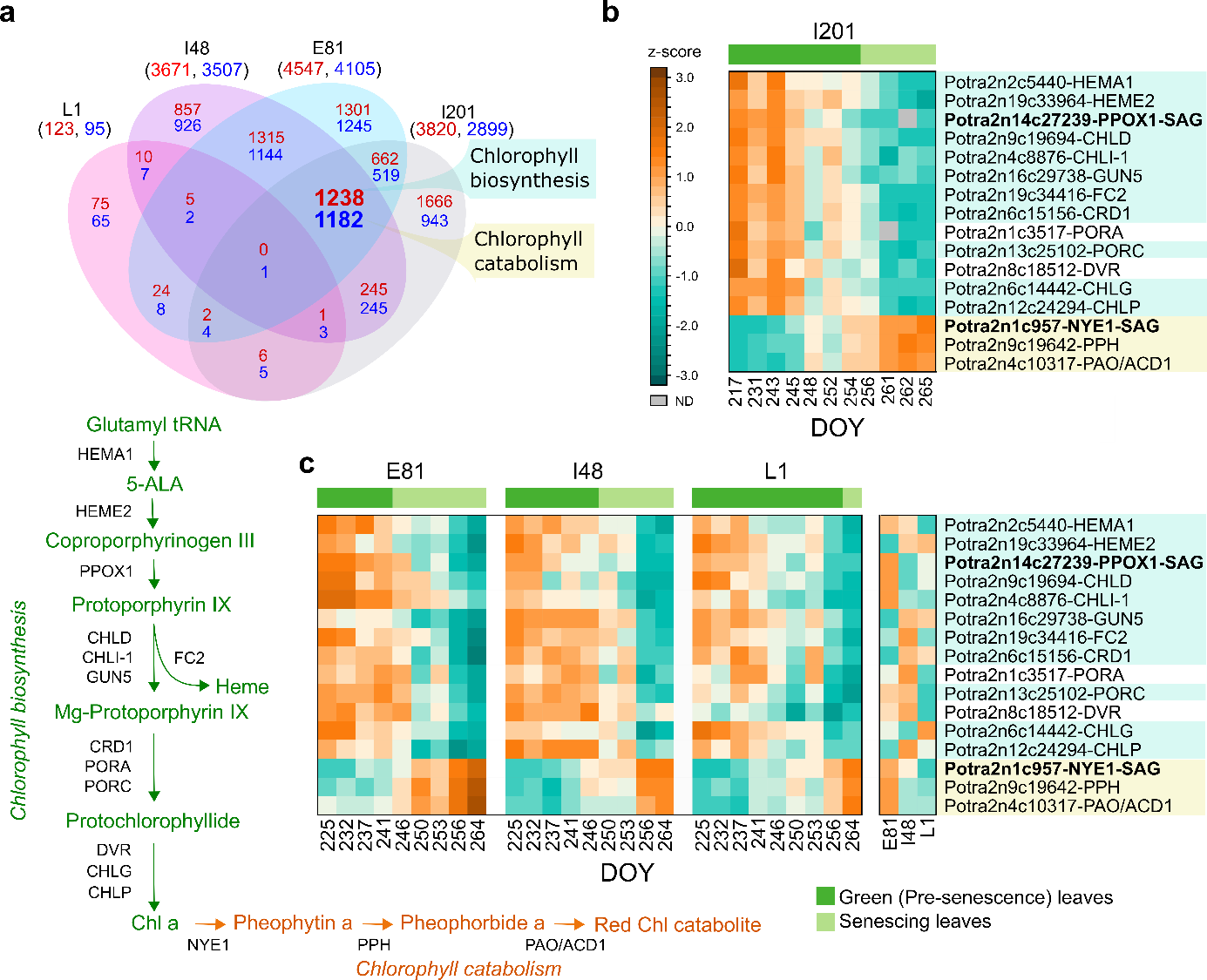
*

**Fig. S15.** **Expression of genes involved in chlorophyll metabolism during autumn.**

Venn diagram depicts the overlap of genes with significant correlation (positive-red, negative-blue) with chlorophyll content index (CCI) in aspen genotypes over the two study years (**a**). The expression patterns of genes involved in chlorophyll biosynthesis and catabolism (based on Arabidopsis) are presented for genotype I201 in 2011 (**b**) and for the three SwAsp genotypes in autumn 2018 (**c**). Mean expression values are normalised to z-scores across time points in autumn (right panels) and across genotypes (left panel). Genes identified as senescence associated genes (SAGs) in both study years irrespective of genotype are in bold and genes with significant correlation with CCI in E81, I48 and I201 are highlighted. Source data are provided as Source Data files.


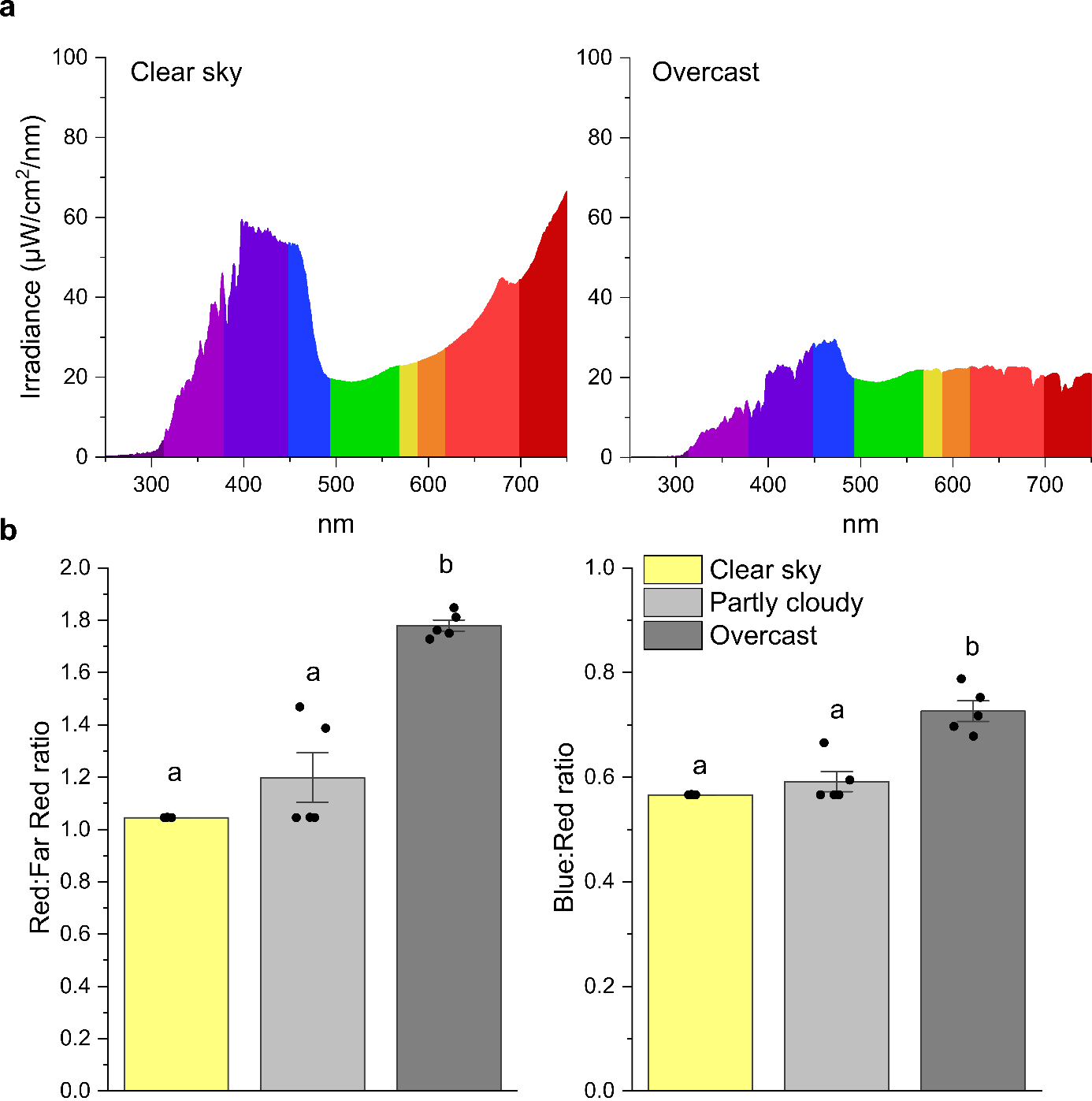


**Fig. S16.** **Solar spectral quality in Umeå, Sweden, in autumn 2020**.

Representative light spectrum on a sunny day with clear sky and on a fully overcast cloudy day (**a**). Red:far red and blue:red ratios on sunny days with clear or almost clear sky (n=3), partly cloudy days (n=5) and almost or fully overcast cloudy days (n=5) in autumn (**b**). Each data point is a mean of 3-6 spectra measured around noon. Data are mean ± SE. The effects of weather conditions were significant for R:FR (F=34.554, *P*-value=3.2e-5) and for B:R (F=21.456, *P*-value=2.4e-4). Different letters represent significantly different means (ANOVA Tukey post hoc *P-*value <0.05 two-sided). Source data are provided as Source Data files.


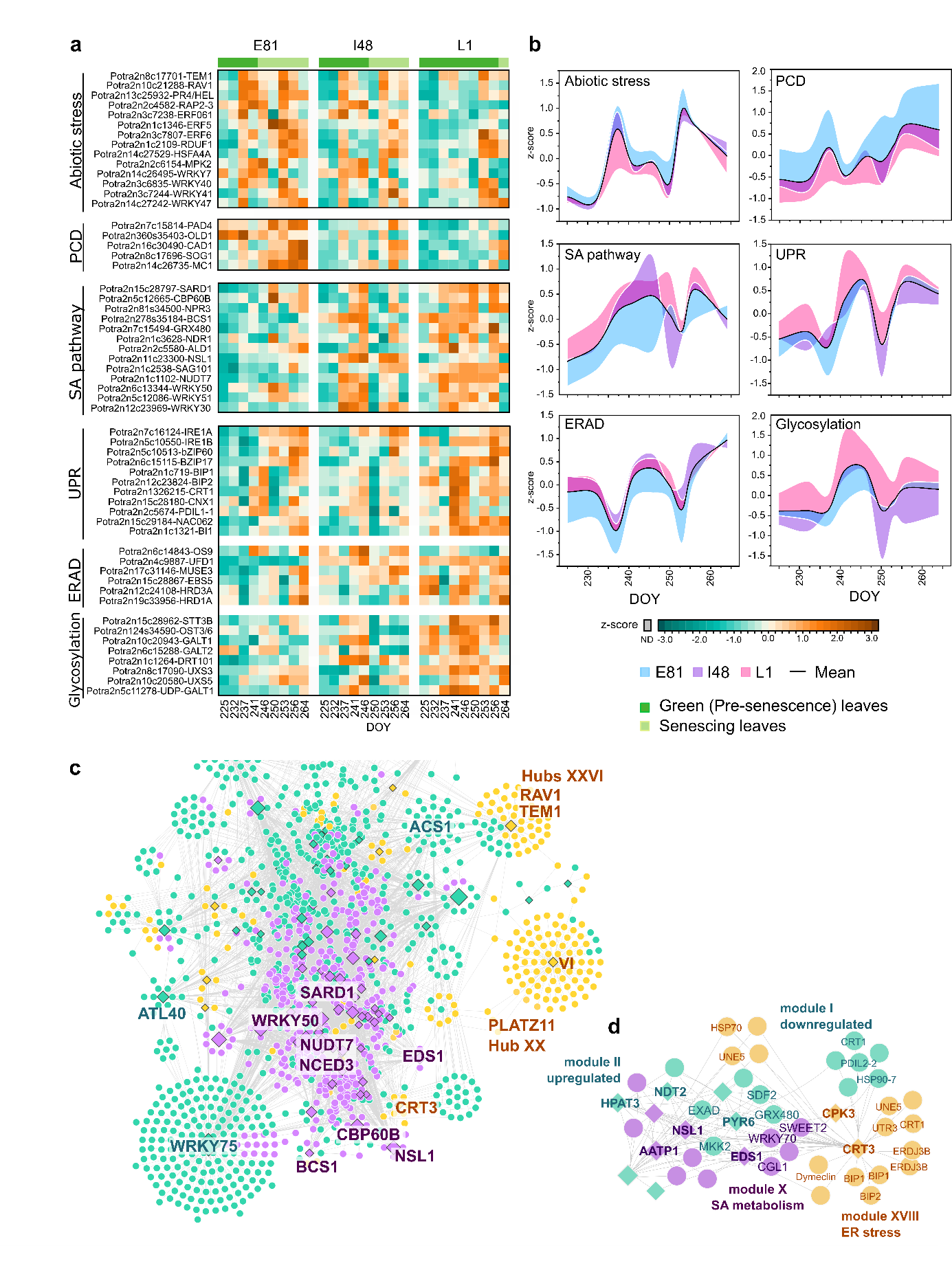


**Fig. S17.** **Expression patterns of genes involved in abiotic stress, programmed cell death (PCD), salicylic acid (SA) pathway and endoplasmic reticulum (ER) stress responses in three SwAsp genotypes in autumn 2018.** Data in the heatmaps are z-score normalized levels (**a**). Overview data (**b**) are averaged z-scores of multiple genes, expressed relative to the overall mean, the deviation of genotypic mean represented by a coloured area. Data are mean of n=2-3 in each time point per genotype. Gene co-expression network displays the close neighbours of genes with positive correlation with SA levels (**c**) and a subnetwork (**d**) displays the potential mediators between modules I and II in cascade-1 and SA- (X) and ER stress-associated (XVIII) gene modules. The network contains edges with Pearson correlation coefficient *r* > 0.6 and FDR adjusted *P*-value <0.05 (two-sided) in the three SwAsp networks. Nodes are coloured based on the associated cascade and the diamond shape represents genes with consistent positive correlation with SA levels. Source data are provided as Source Data files.


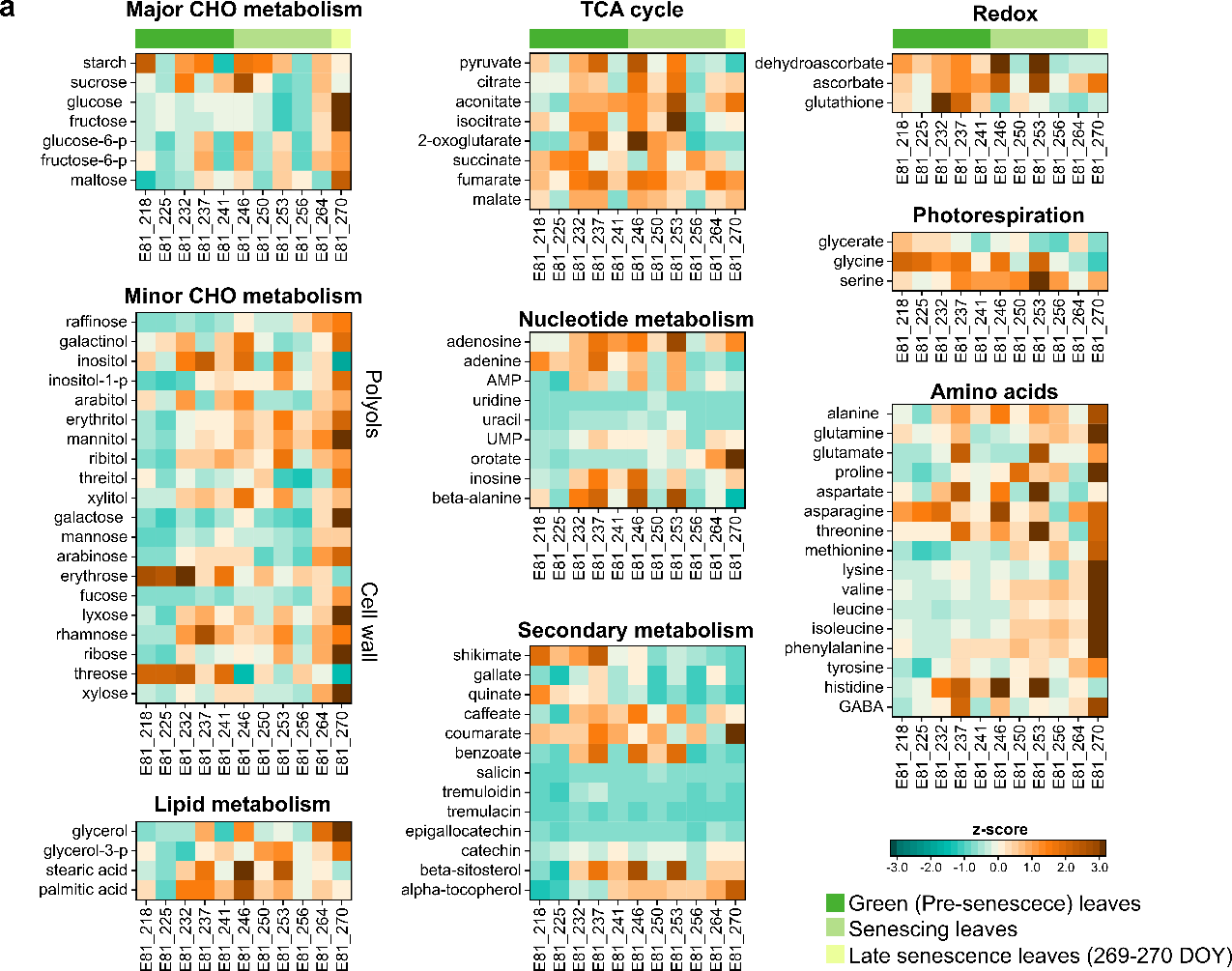


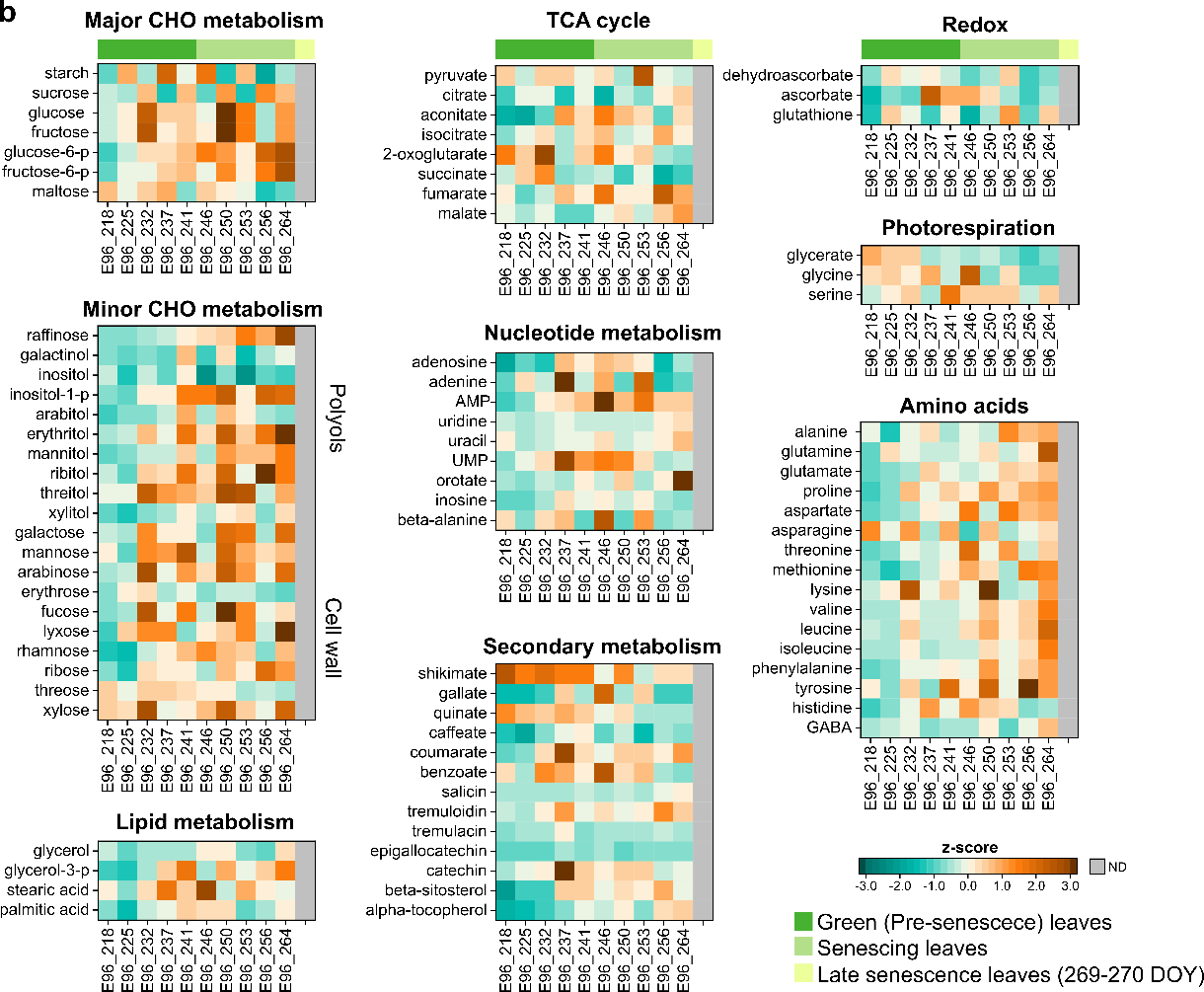


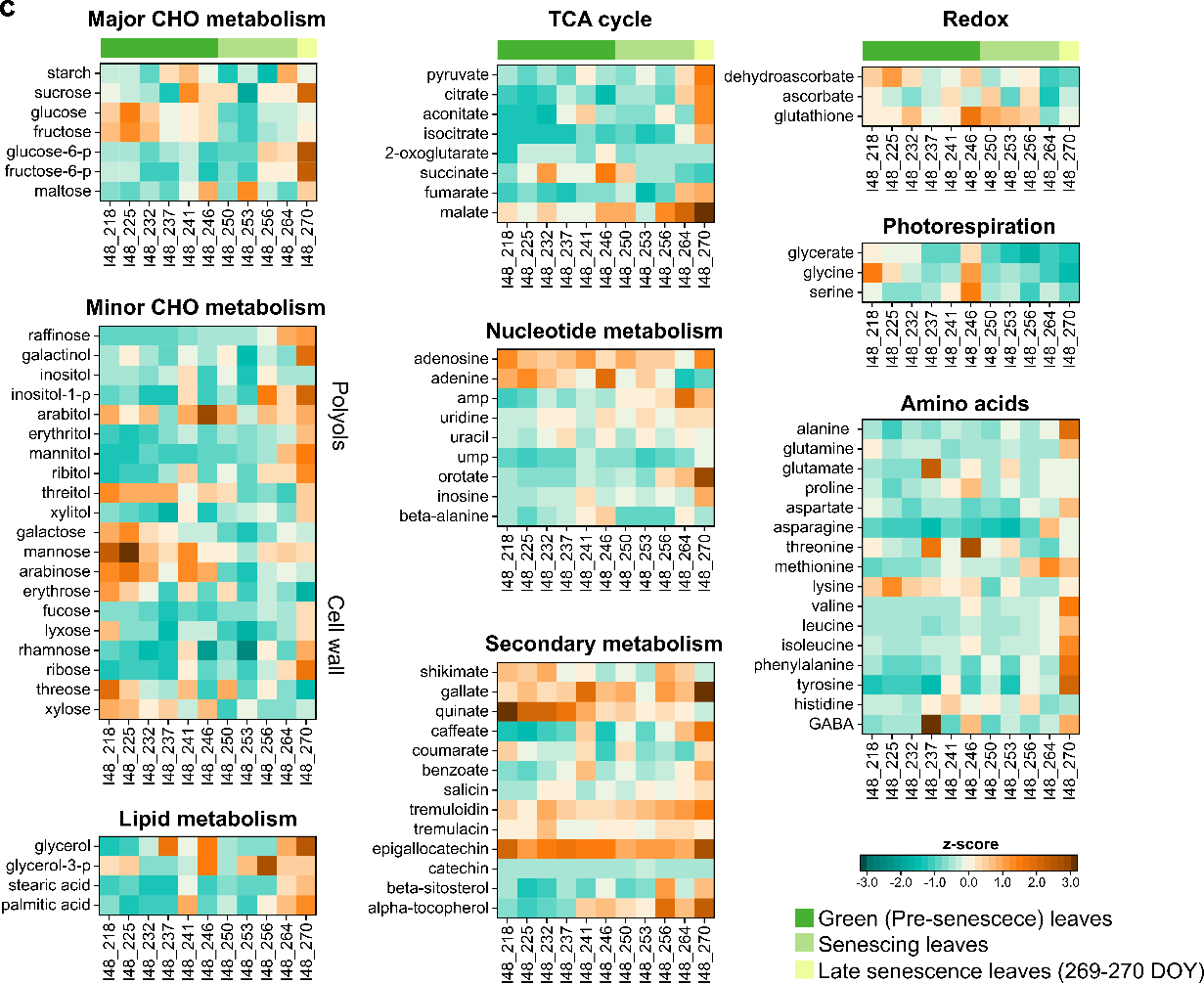


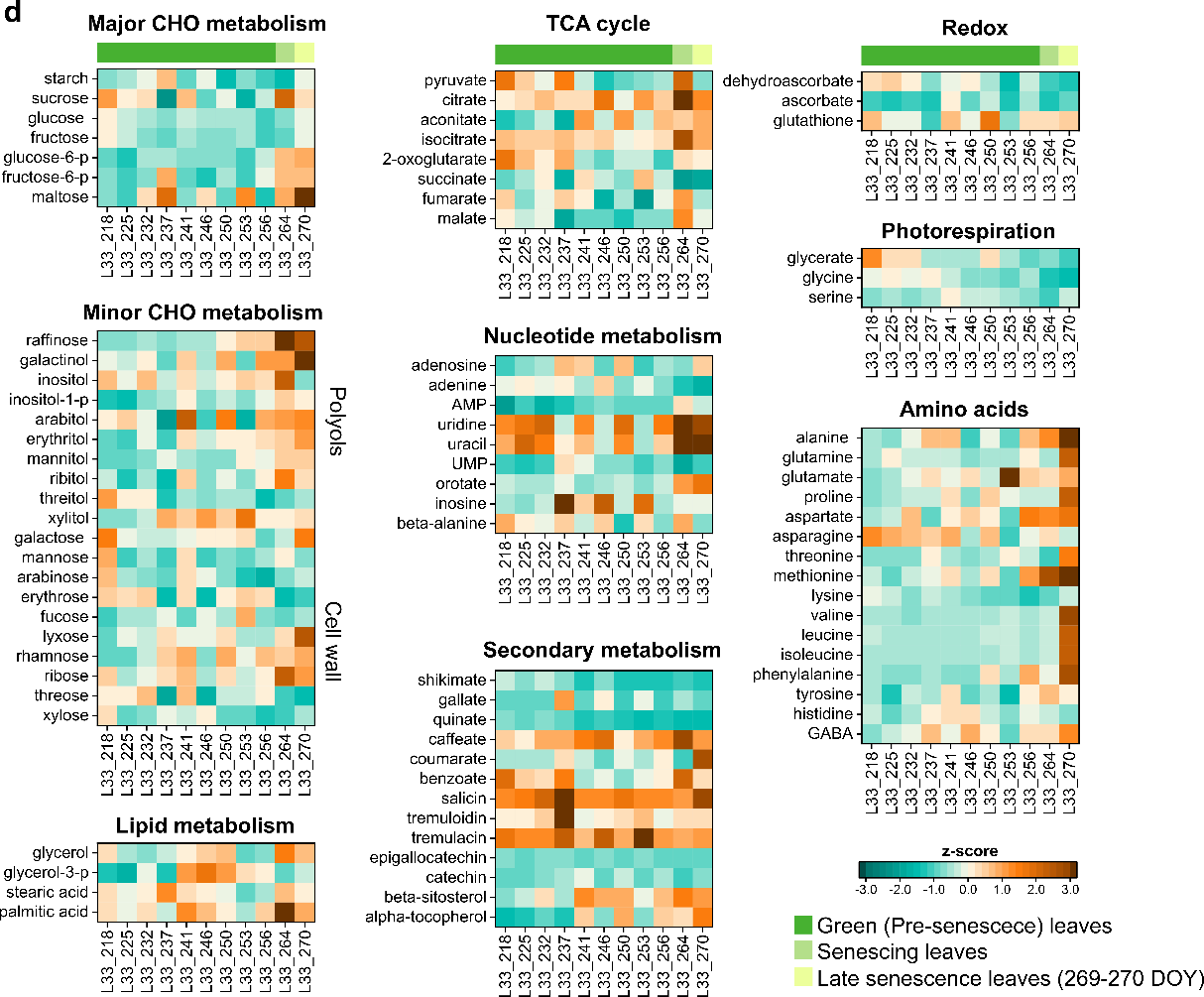


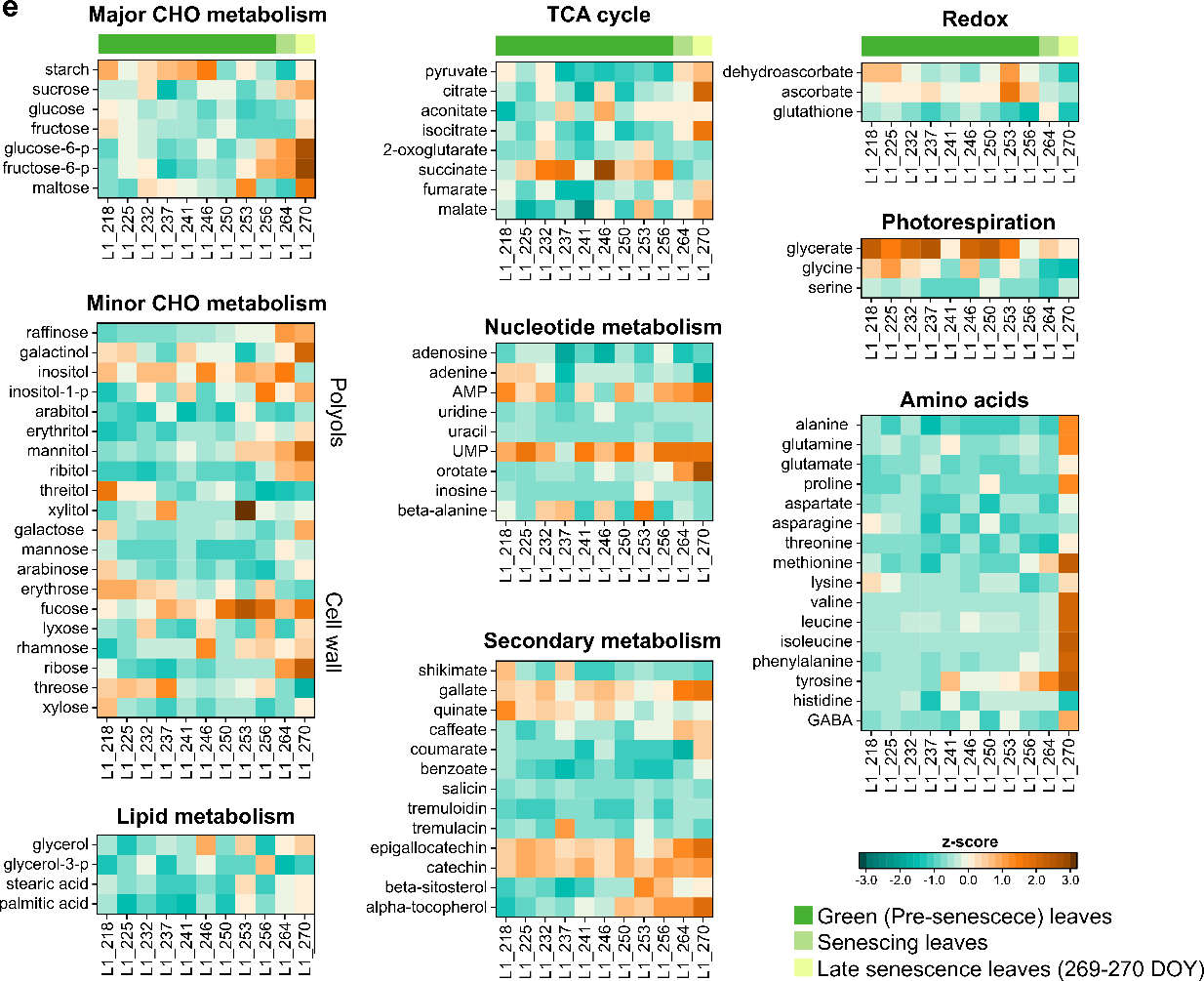


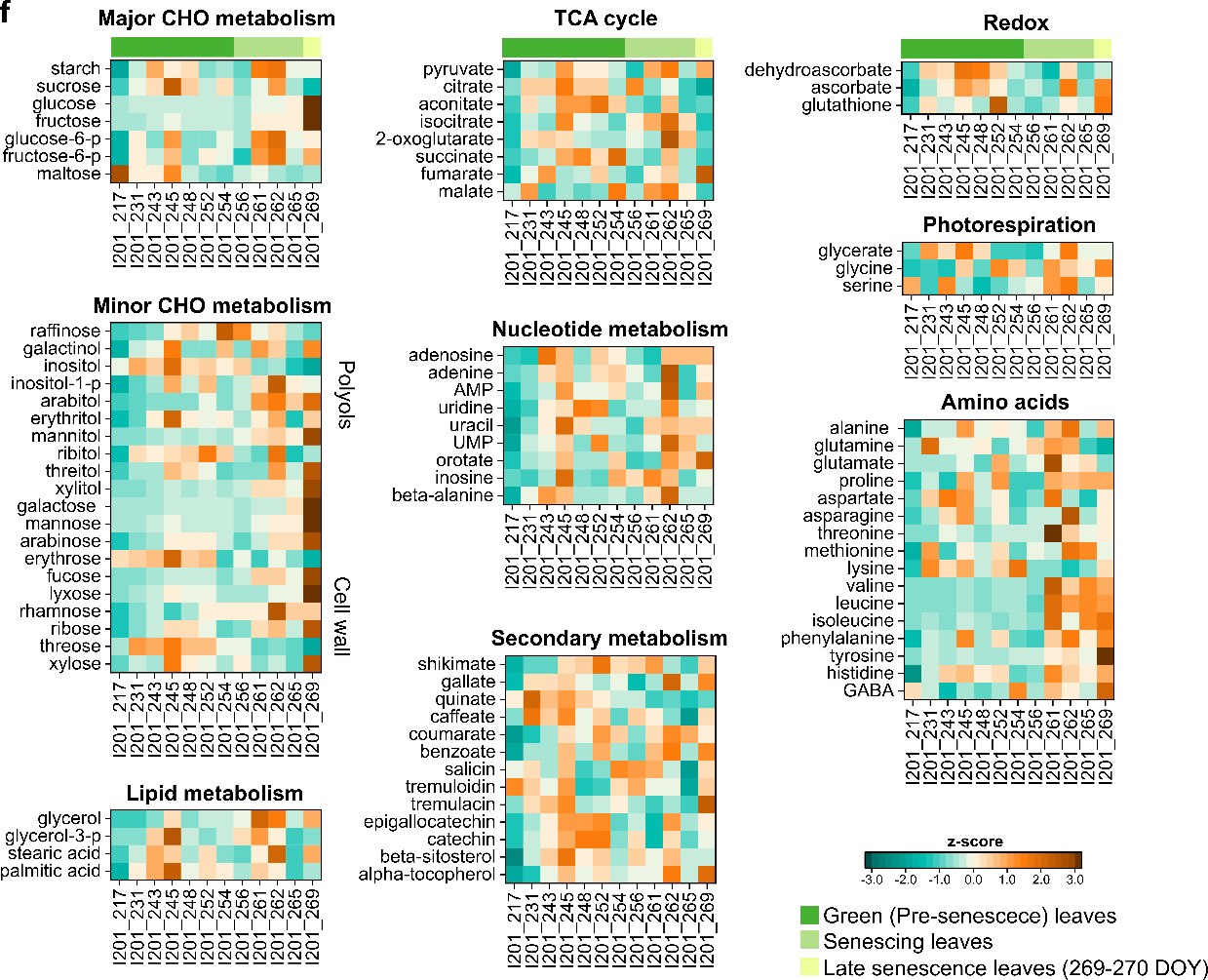


**Fig. S18. Overview of metabolite responses in aspen leaves in autumn.** Heatmaps display the mean metabolite levels normalized to z-scores in the leaves of five aspen genotypes, E81 (**a**), E96 (**b**), I48 (**c**), L33 (**d**) and L1 (**e**) in autumn 2018 (218-270 DOY), and in the leaves of genotype I201 (**f**) in autumn 2011 (217-269 DOY). Source data are provided as Source Data files.


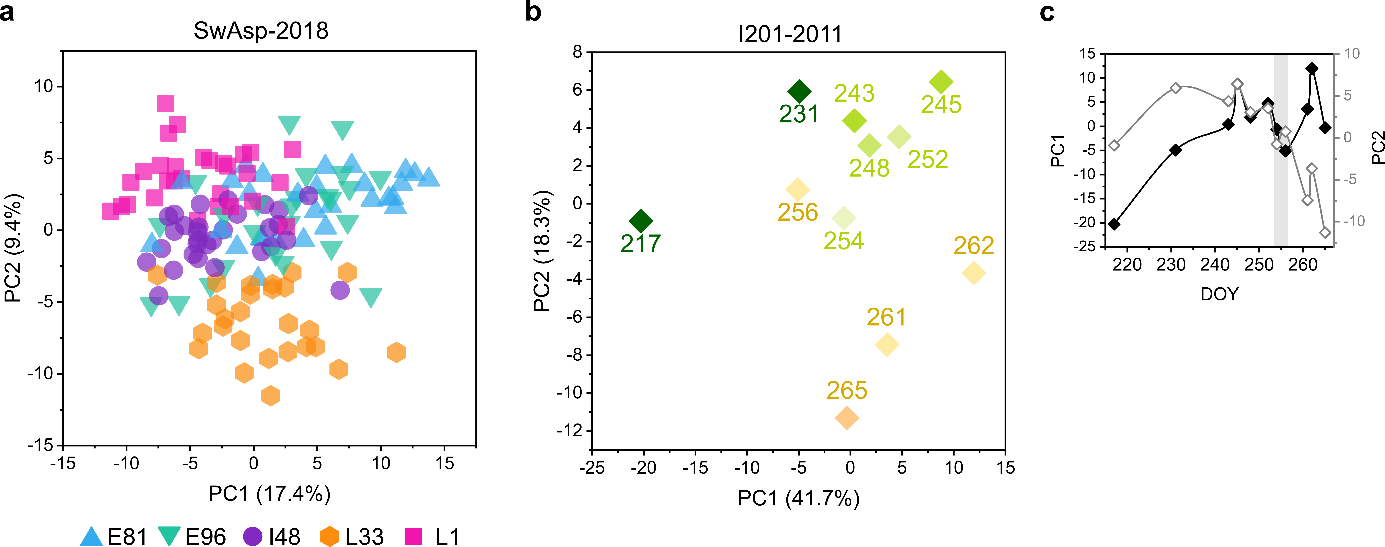


**Fig. S19.** **Principal component analysis (PCA) of metabolite marker profiles in five SwAsp genotypes in autumn 2018 and in genotype I201 in 2011.**

PCA scores plot of metabolite markers are coloured based on five SwAsp genotypes in autumn 2018 (**a**), n=25 in E96, n=26 in L33, n=29 in E81 and L1, and n=30 in I48. The second principal component (PC2) separates two late-senescing genotypes from each other and from early- and intermediate-senescing genotypes (**a**). PCA scores plot (**b**) and time-dependent plot of PC scores in I201 in autumn 2011 (**c**), n=1 in each time point. The two significant PCs show time-dependent variation in genotype I201 during autumn (**b, c**). The day of the year (DOY) is presented beside the samples (**b**) and the shaded vertical line represents senescence onset (**c**). Source data are provided as Source Data files.

**
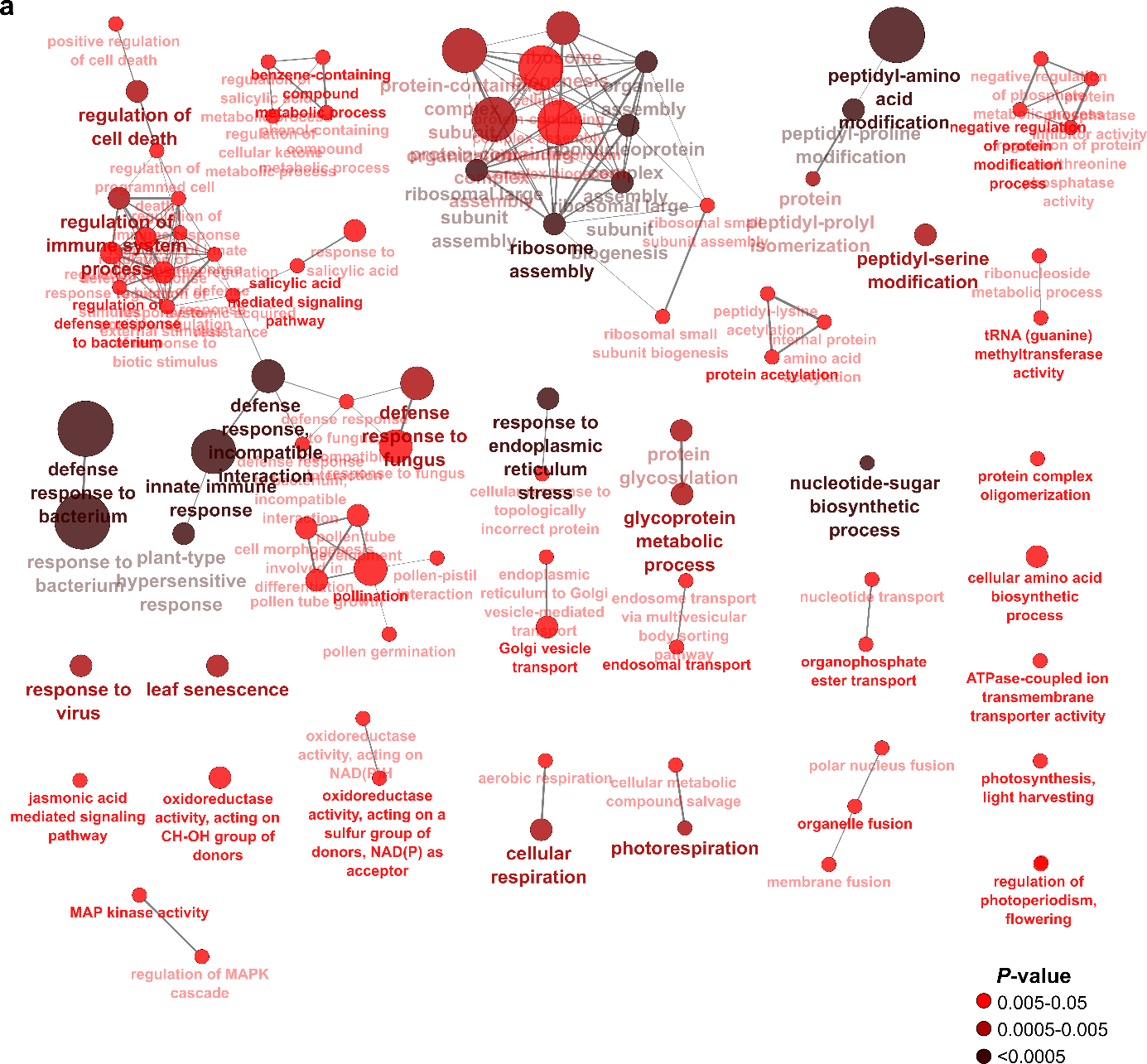
**

**
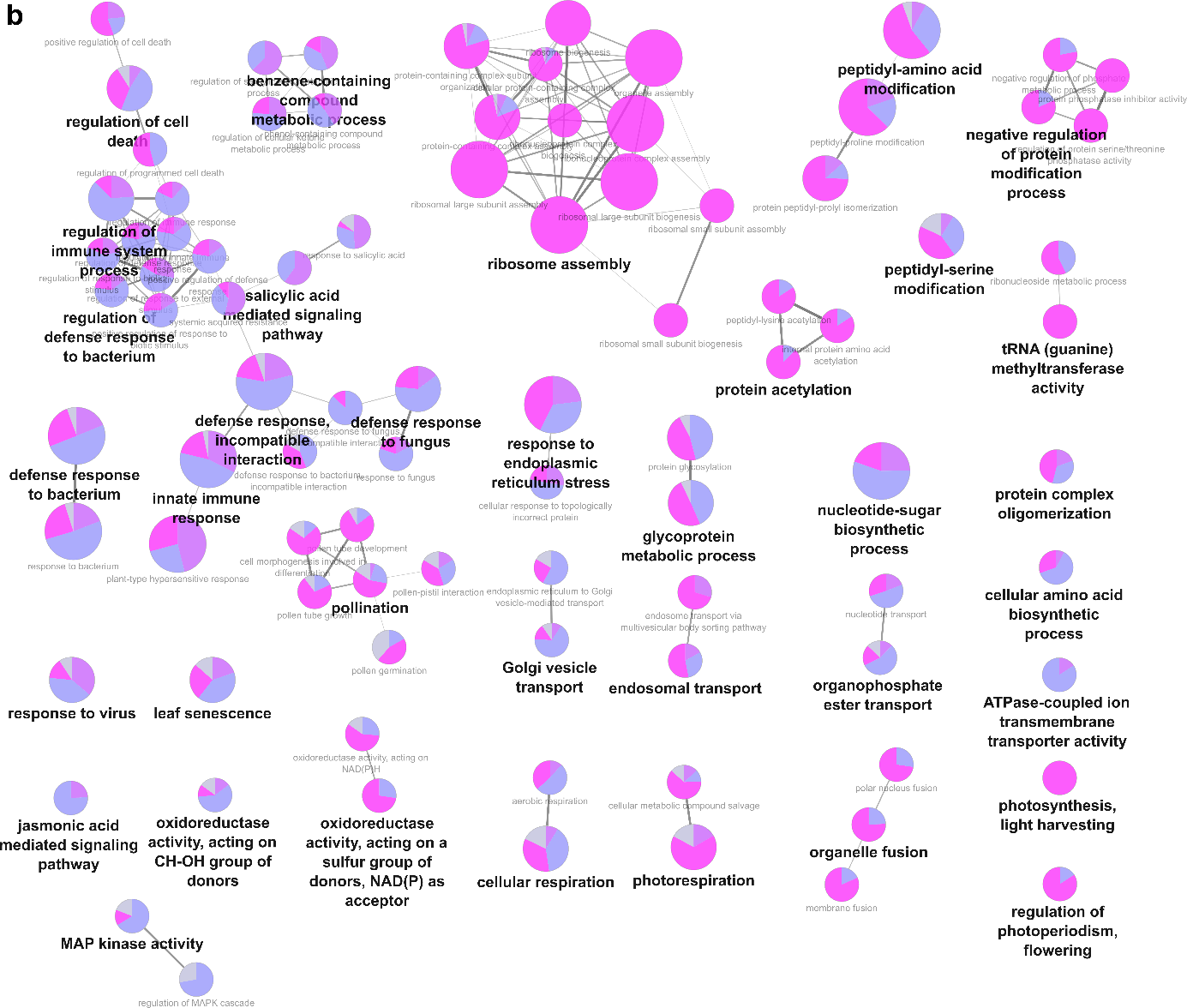
**

**
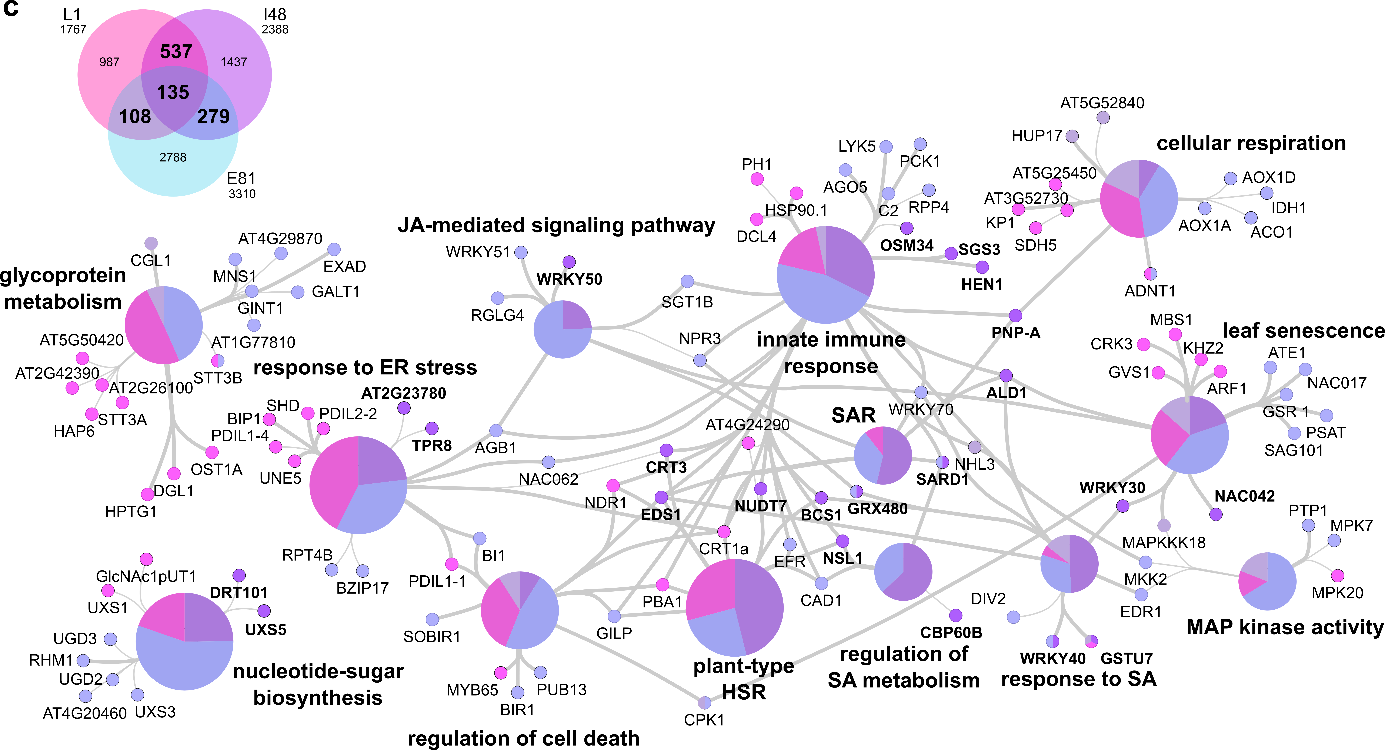
**

**Fig. S20.** **Gene Ontology (GO) term enrichment analysis of genes with positive correlation with salicylic acid (SA).**

The genes with significant positive correlation with SA at least in two SwAsp genotypes were included in the analysis. GO term enrichment analysis and network were performed with Cytoscape ClueGO and CluePedia applications. The nodes are coloured based on the *P*-value, a darker shade depicting higher significance (**a**) and the size of the GO term node is proportional to the number of matched genes. The nodes are coloured according to the proportion of genes with positive correlation with SA levels (**c**) between genotypes as depicted in the Venn diagrams (**c**). GO term network displays the connection of genes among the selected enriched biological processes (**c**). The genes with consistent positive correlation with SA levels (**c**) in all three SwAsp genotypes are in bold. The details of the correlation results are in Supplementary Data 15.


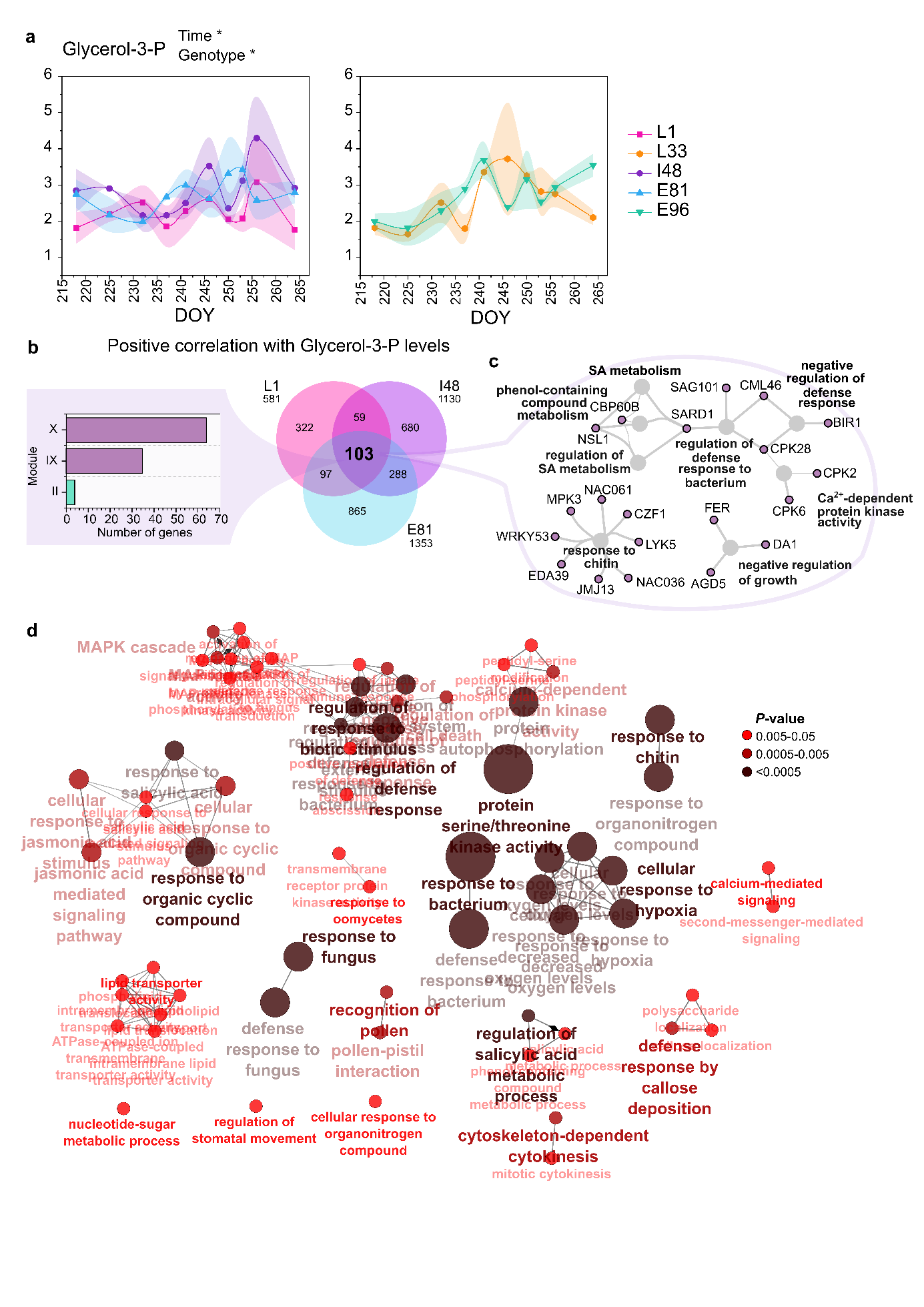


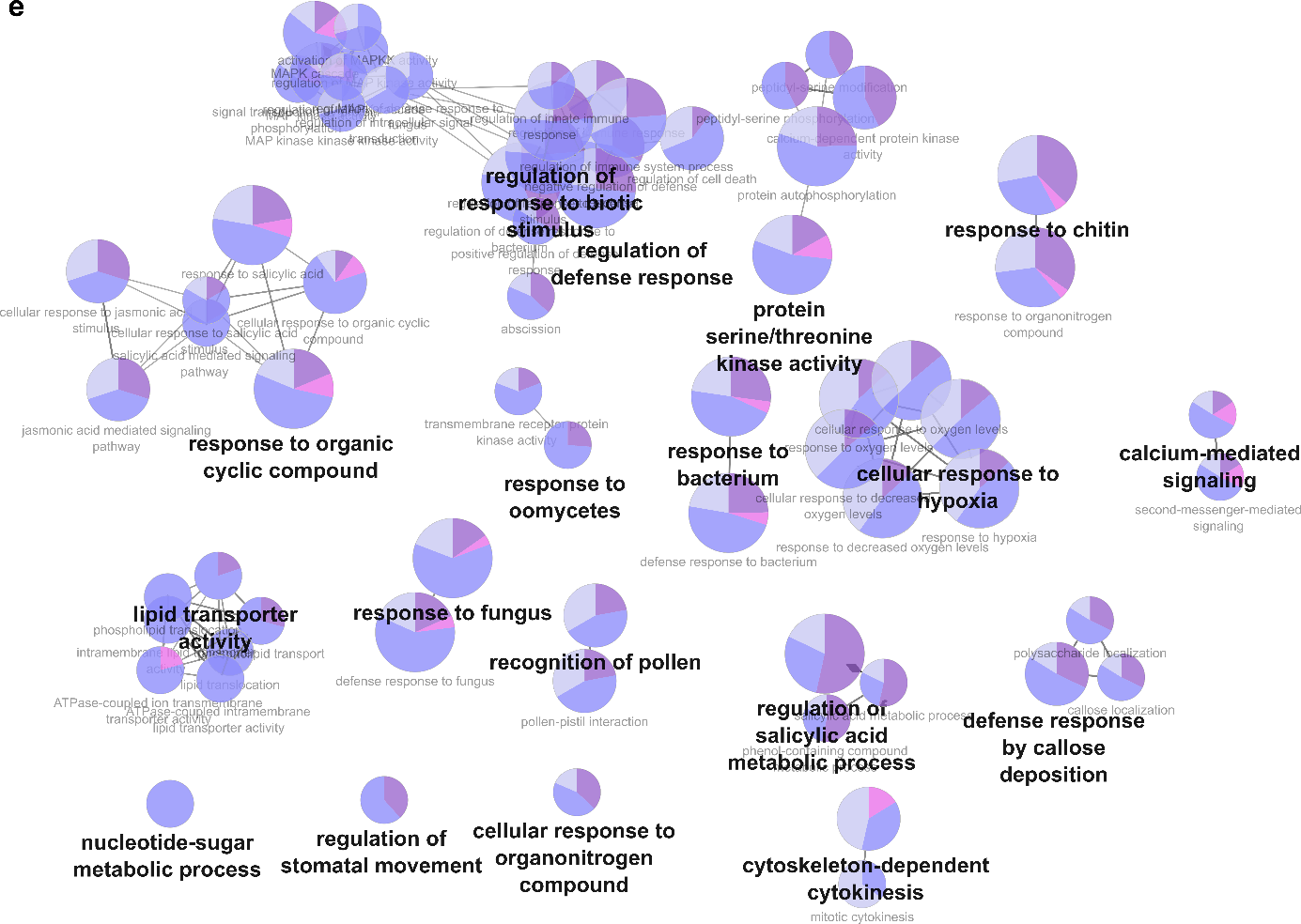


**Fig. S21. Gene Ontology (GO) term enrichment analysis of genes with positive correlation with glycerol-3-phosphate (Glycerol-3-P) levels.**

Glycerol-3-P levels in five aspen genotypes during autumn (**a**). The effects of time and genotype were tested with two-way ANOVA (Supplementary Data 14). Data are mean ± SE (shadowed area), n=2-3 in each time point per genotype, see details in Supplementary Data 14. Bar plot displays the number of genes with significant positive correlation with glycerol-3-P levels in the three SwAsp genotypes selected for transcriptomics (**b**). Venn diagram shows the number and overlap of genes with positive correlation with glycerol-3-P levels in the SwAsp genotypes (**b**). GO term network displays significantly enriched terms for biological processes in the gene set and the associated genes (**c**). Extended GO term enrichment analysis of genes that showed significant correlation with glycerol-3-P at least in two SwAsp genotypes (**d, e**). The nodes are coloured based on the *P*-value, darker shades depicting higher significance (**d**) and the size of the GO term node is proportional to the number of matched genes. The nodes are coloured according to the proportion of genes with positive correlation with glycerol-3-P levels (**e**) between genotypes as depicted in the Venn diagram (**b**). The details of correlation results are in Supplementary Data 15. Source data are provided as Source Data files.


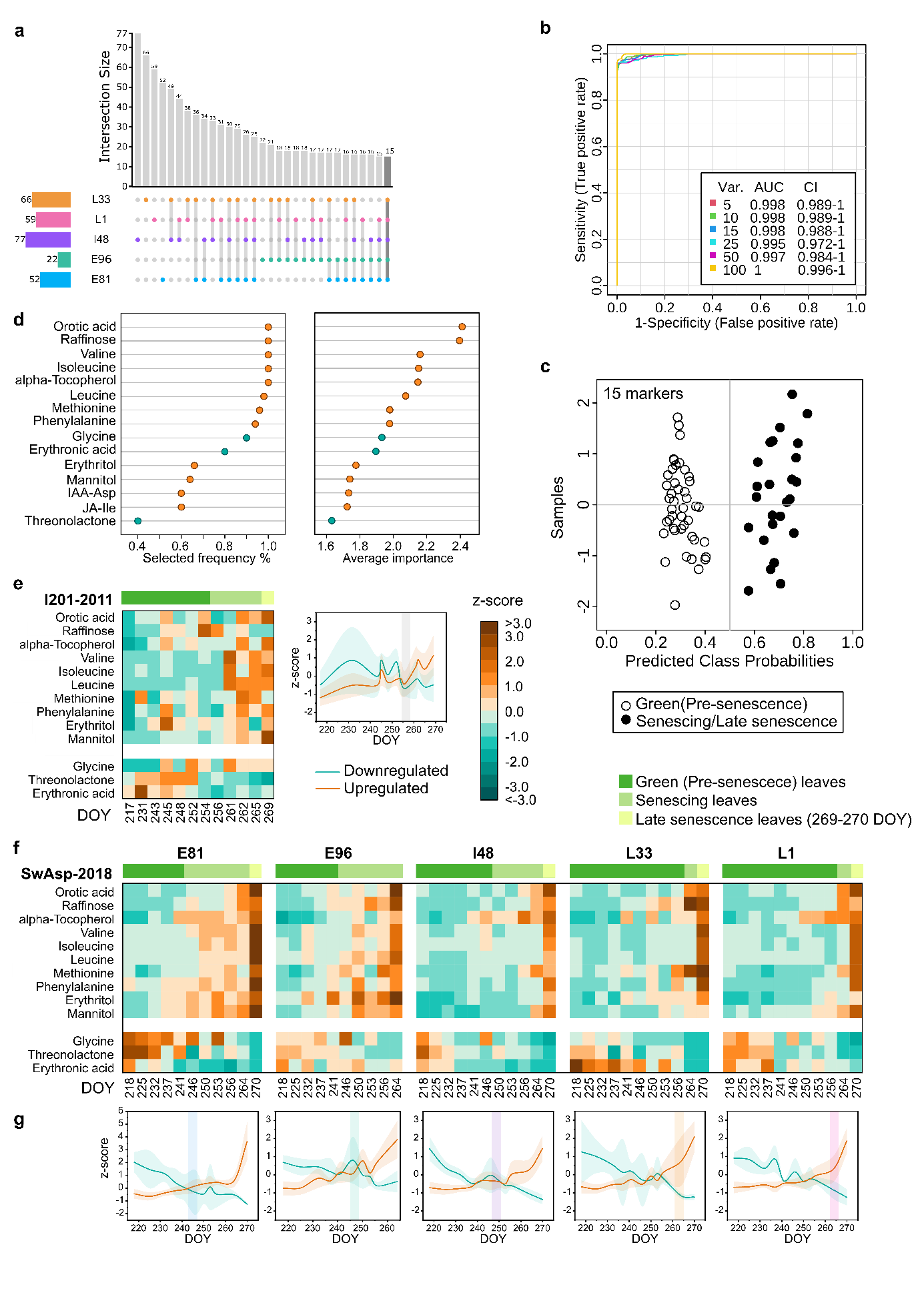


**Fig. S22. Autumn senescence is accompanied by a comprehensive shift in metabolite profile and altered levels of senescence-associated metabolic markers.**

UpSet-plot displays the intersection of metabolite markers with significantly different levels (t-test, FDR adjusted *P*-value <0.05, two-sided) between green (pre-senescence) and senescing/late senescence leaves in five SwAsp genotypes in autumn 2018 (**a**). 15 metabolite markers were shared in all and another 15 in four out of five genotypes. AUC (Area Under the Curve) of multivariate Partial Least-Squares Discriminant Analysis (PLS-DA) models separating samples from green (pre-senescence) and senescing/late senescence leaves based on 5-100 metabolite markers (**b**). Class prediction for samples in senescence groups based on top 15 metabolite markers (training set, **c**). Selected frequency and average importance of the top 15 metabolite senescence markers (**d**). The levels of 13 common metabolite markers in the leaves of genotype I201 during autumn 2011 (**e**) and in SwAsp genotypes in autumn 2018 (**f**). The shift in metabolite markers presented in terms of mean z-score of up- or down-regulated markers ± SD (shadowed area) in respect to the start of leaf senescence in a particular genotype (shadowed line) (**e**, **g**). Data in the heatmaps are z-score normalized levels, mean n=2-3 for SwAsp and n=1 for genotype I201 in each time point. See the details of the statistical results in Supplementary Data 16. Source data are provided as Source Data files.


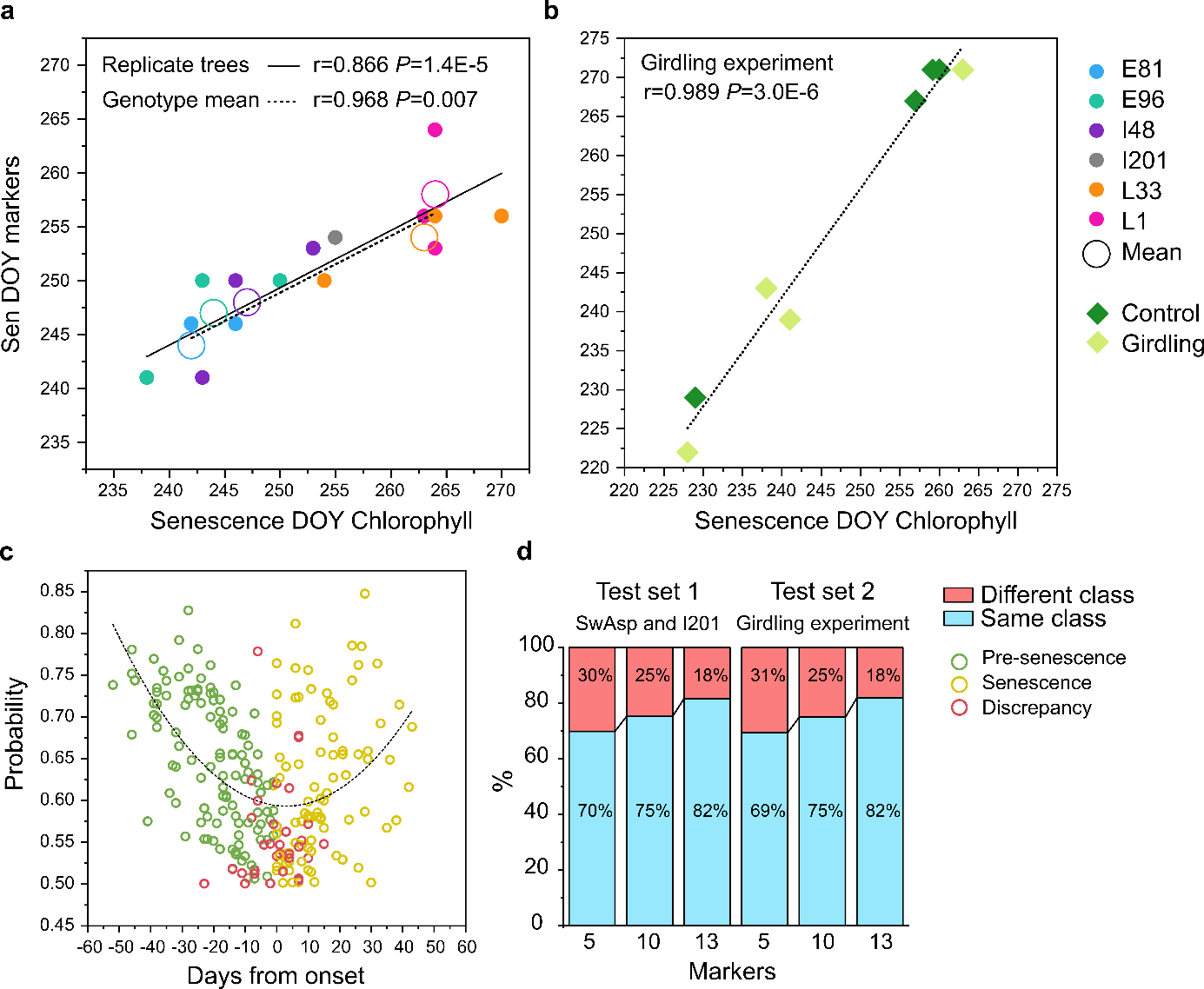


**Fig. S23.** **Chlorophyll content index and metabolic markers give comparable estimates for the date of autumn senescence onset.**

The correlation (Pearson *r,* two-sided *P*-value) between senescence onset date (the day of the year, DOY) estimated based on the start of rapid chlorophyll depletion and 13 metabolic markers in SwAsp in 2018 and in genotype I201 in autumn 2011 (**a**), and in the girdling experiment (**b**, Lihavainen et al. ^16^). Samples collected around the estimated onset date (based on chlorophyll) displayed the most discrepancy in the classification of samples as green (pre-senescence) or senescing/late senescence leaves with the two methods (**c**). The similarity of the class prediction by the two methods increased by the increasing number of metabolic markers used in the model from five to 13 (**d**). The top metabolic markers were defined based on Partial Least Squares Discriminant Analysis (PLS-DA, MetaboAnalyst). See details of the markers and model performance in Fig. S20 and the comparison of results between different models and senescence proxies in Supplementary Data 16.

**
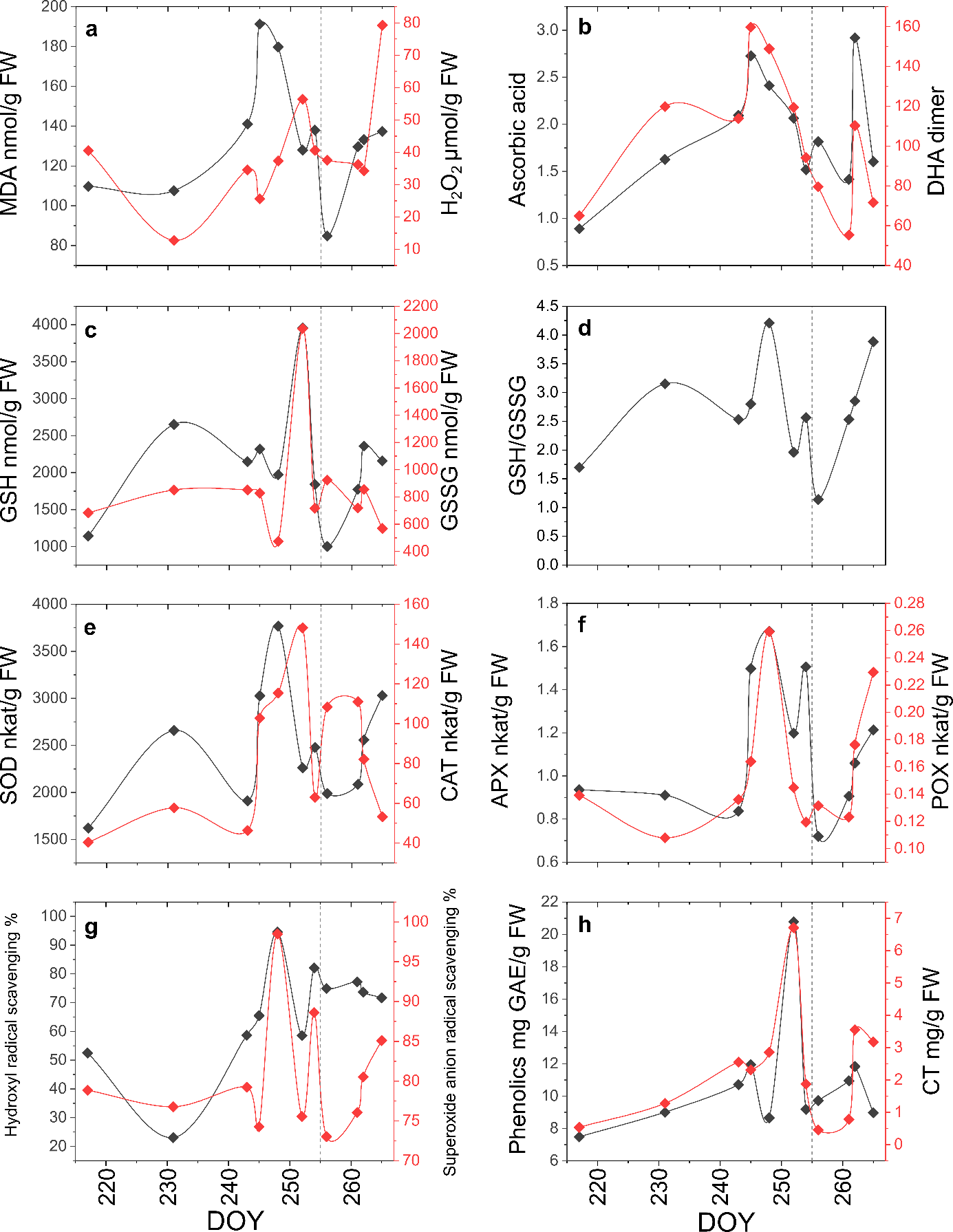
**

**Fig. S24.** **Signalling molecule levels and enzymatic and non-enzymatic antioxidant systems in genotype I201 during autumn 2011.**

The levels of reactive oxygen species (ROS), signalling molecules and metabolic antioxidants: malondialdehyde (MDA nmol/g FW, **a**), hydrogen peroxide (H_2_O_2_ µmol/g FW, **a**), ascorbic acid (**b**) and dehydroascorbic acid (DHA, **b**) dimer (arbitrary units), glutathione (GSH, GSSG nmol/g FW, **c**) and GSH/GSSG ratio (**d**), the activities (nkat/g FW) of superoxide dismutase (SOD, **e**) and catalase (CAT, **e**), ascorbate peroxidase (APX, **f**) and pyrogallol peroxidase (POX, **f**), hydroxy radical (^•^OH**)** and superoxide anion radical (O₂·⁻ ) scavenging capacities (%) of methanolic leaf extracts (**g**), and the total abundance of phenolics (mg GAE /g FW) and condensed tannins (CT mg/g FW) in I201 in autumn 2011 (**h**). Data in the line graphs are mean of two technical replicates from one tree, n=1. Vertical dashed line represents the estimated date of senescence onset. Source data are provided as Source Data files.


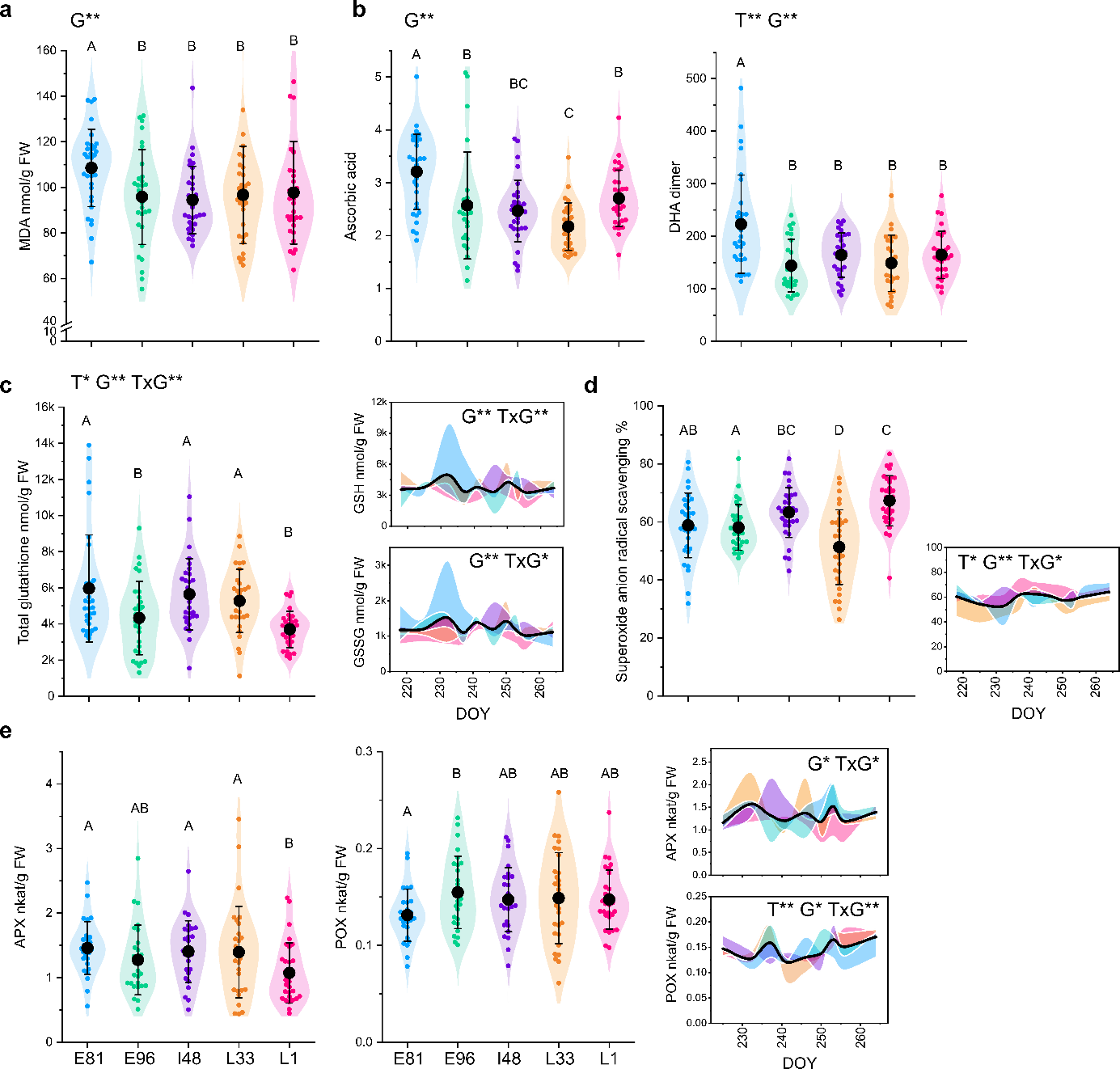


**Fig. S25. Signalling molecule levels and enzymatic and non-enzymatic antioxidant systems in five SwAsp genotypes during autumn 2018.**

The levels of malondialdehyde (MDA nmol/g FW, **a**), ascorbic acid metabolites (ascorbic acid and dehydroascorbic acid arbitrary units, **b**), total glutathione and temporal patterns of GSH and GSSG (nmol/g FW, **c**), superoxide anion radical (O₂·⁻) scavenging capacity (%) of methanolic leaf extracts (**d**), and the activities of ascorbate peroxidase (APX) and pyrogallol peroxidase (POX) in the SwAsp genotypes (**e**). Metabolite levels and scavenging activities through the time course in each genotype (mean, n=3) are presented relative to the overall mean, coloured area presents the difference between the genotype mean and the overall mean. Violin plots display all data points during the study period in each genotype, black points represent the mean and whiskers standard deviation (± SD). The effects of time, genotype and their interaction were tested with two-way ANOVA (FDR adjusted *P*-value <0.05*, <0.01**). The different letters mark significantly different means between the genotypes (Fisher’s Least Significant Difference LSD, *P*-value <0.05, two-sided). See the detailed statistical results in Supplementary Data 14. Source data are provided as Source Data files.

**Supplementary References**

1. Ivanov Dobrev, P. & Kamı́nek, M. Fast and efficient separation of cytokinins from auxin and abscisic acid and their purification using mixed-mode solid-phase extraction. *J. Chromatogr. A* **950**, 21–29 (2002).

2. Floková, K. *et al.* UHPLC–MS/MS based target profiling of stress-induced phytohormones. *Phytochemistry* **105**, 147–157 (2014).

3. Svačinová, J. *et al.* A new approach for cytokinin isolation from Arabidopsis tissues using miniaturized purification: pipette tip solid-phase extraction. *Plant Methods* **8**, 17 (2012).

4. Gullberg, J., Jonsson, P., Nordström, A., Sjöström, M. & Moritz, T. Design of experiments: an efficient strategy to identify factors influencing extraction and derivatization of Arabidopsis thaliana samples in metabolomic studies with gas chromatography/mass spectrometry. *Anal. Biochem.* **331**, 283–295 (2004).

5. Langfelder, P. & Horvath, S. WGCNA: an R package for weighted correlation network analysis. *BMC Bioinformatics* **9**, 559 (2008).

6. Sundell, D. *et al.* The Plant Genome Integrative Explorer Resource: PlantGenIE.org. *New Phytol.* **208**, 1149–1156 (2015).

7. Raudvere, U. *et al.* g:Profiler: a web server for functional enrichment analysis and conversions of gene lists (2019 update). *Nucleic Acids Res.* **47**, W191–W198 (2019).

8. Shannon, P. *et al.* Cytoscape: A Software Environment for Integrated Models of Biomolecular Interaction Networks. *Genome Res.* **13**, 2498–2504 (2003).

9. Bindea, G. *et al.* ClueGO: a Cytoscape plug-in to decipher functionally grouped gene ontology and pathway annotation networks. *Bioinformatics* **25**, 1091–1093 (2009).

10. Bindea, G., Galon, J. & Mlecnik, B. CluePedia Cytoscape plugin: pathway insights using integrated experimental and in silico data. *Bioinformatics* **29**, 661–663 (2013).

11. Li, Z. *et al.* LSD 3.0: a comprehensive resource for the leaf senescence research community. *Nucleic Acids Res.* **48**, D1069–D1075 (2020).

12. Lu, H., Gordon, M. I., Amarasinghe, V. & Strauss, S. H. Extensive transcriptome changes during seasonal leaf senescence in field-grown black cottonwood (Populus trichocarpa Nisqually-1). *Sci. Rep.* **10**, 6581 (2020).

13. Fracheboud, Y. *et al.* The Control of Autumn Senescence in European Aspen. *Plant Physiol.* **149**, 1982–1991 (2009).

14. Edlund, E., Novak, O., Karady, M., Ljung, K. & Jansson, S. Contrasting patterns of cytokinins between years in senescing aspen leaves. *Plant Cell Environ.* **40**, 622–634 (2017).

15. Michelson, I. H. *et al.* Autumn senescence in aspen is not triggered by day length. *Physiol. Plant.* **162**, 123–134 (2018).

16. Lihavainen, J. *et al.* Stem girdling affects the onset of autumn senescence in aspen in interaction with metabolic signals. *Physiol. Plant.* **172**, 201–217 (2021).

**Supplementary Note 1**

A list of Supplementary Data and Source Data files containing the source data of the figures:

Fig. 1b-f: Source_Data_1

Fig. 2a-b: Source_Data_1

Fig. 2c: Source_Data_3: DESeq_results: LRT

Fig. 2f: Supplementary Data 2

Fig. 2g-h: Source_Data_3

Fig. 3a: Source_Data_2

Fig. 3b: Supplementary Data 4 Table h

Fig. 4: Supplementary Data 14 Table b (Mean and SE), Table c (statistics)

Fig. 5a: Source_Data_3

Fig. 5b: Supplementary Data 15 Table a-c

Fig. 6a-b: Source_Data_1

Fig. 6c: Source_Data_4

Fig. 6d: Supplementary Data 14 Table b (Mean, SE), Source Data 2 (network)

Fig. 6e: Supplementary Data 14 Table c

Fig. 6f: Source_Data_3

Fig. 7a: Source_Data_1

Fig. 7b-f: Source_Data_4, Supplementary Data 14 Table b (Mean and SE), Table c (statistics)

Fig. 7g: Source_Data_3

Fig. S3: Source_Data_1

Fig. S4: Source_Data_3: DESeq_results

Fig. S9: Source_Data_4

Fig. S11a: Source_Data_2

Fig. S11b: Supplementary Data 4 Table h

Fig. S11c: Supplementary Data 4 Table a-b

Fig. S11d: Supplementary Data 4 Table j

Fig. S13: Source_Data_2 (network), Source_Data_3 (gene expression)

Fig. S15: Supplementary Data 9 and 13, Source_Data_3 (gene expression)

Fig. S16: Source_Data_1

Fig. S17: Source_Data_2 (network), Source_Data_3 (gene expression)

Fig. S18: Supplementary Data 14 Table b (Mean, SE)

Fig. S19: Source_Data_1

Fig. S20: Supplementary Data 15 Table a-c

Fig. S21a: Supplementary Data 14 Table b (Mean, SE)

Fig. S21b: Supplementary Data 15 Table h

Fig. S22: Supplementary Data 16

Fig. S23: Supplementary Data 16

Fig. S24: Supplementary Data 14 Table b (Mean, SE)

Fig. S25: Source_Data_4, Supplementary Data 14 Table b (Mean, SE), Table c (statistics)

**Supplementary Note 2**


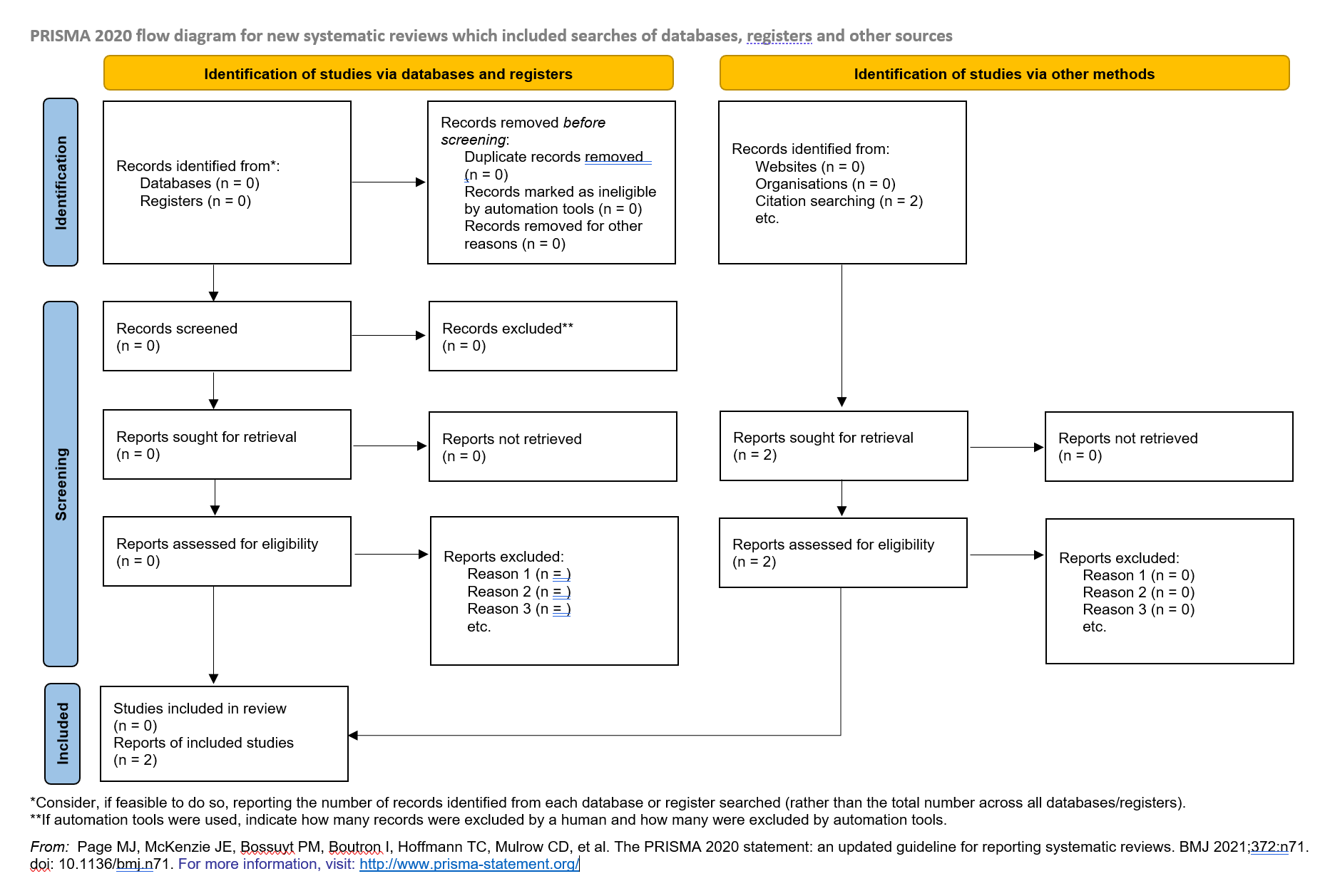

Supplement: Supplementary file 1 — Supplementary Information [file 41467_2023_39564_MOESM1_ESM.docx]
