# Supplementary material for: Salicylic acid metabolism and signalling coordinate senescence initiation in aspen in nature
Source: Nat Commun. 2023 Jul 18;14:4288. doi: 10.1038/s41467-023-39564-5 (PMC10354028; doi:10.1038/s41467-023-39564-5)
Supplement: Supplementary file 2 — Reporting Summary [file 41467_2023_39564_MOESM2_ESM.pdf]

nature portfolio

Corresponding author(s): Stefan Jansson

Last updated by author(s): Jun 13, 2023

Reporting Summary

Nature Portfolio wishes to improve the reproducibility of the work that we publish. This form provides structure for consistency and transparency in reporting. For further information on Nature Portfolio policies, see our Editorial Policies and the Editorial Policy Checklist.

Statistics

For all statistical analyses, confirm that the following items are present in the figure legend, table legend, main text, or Methods section.

n/a	Confirmed	
☐	☒	The exact sample size (*n*) for each experimental group/condition, given as a discrete number and unit of measurement
☐	☒	A statement on whether measurements were taken from distinct samples or whether the same sample was measured repeatedly
☐	☒	The statistical test(s) used AND whether they are one- or two-sided<br>*Only common tests should be described solely by name; describe more complex techniques in the Methods section.*
☐	☒	A description of all covariates tested
☐	☒	A description of any assumptions or corrections, such as tests of normality and adjustment for multiple comparisons
☐	☒	A full description of the statistical parameters including central tendency (e.g. means) or other basic estimates (e.g. regression coefficient) AND variation (e.g. standard deviation) or associated estimates of uncertainty (e.g. confidence intervals)
☐	☒	For null hypothesis testing, the test statistic (e.g. *F*, *t*, *r*) with confidence intervals, effect sizes, degrees of freedom and *P* value noted<br>*Give P values as exact values whenever suitable.*
☒	☐	For Bayesian analysis, information on the choice of priors and Markov chain Monte Carlo settings
☐	☒	For hierarchical and complex designs, identification of the appropriate level for tests and full reporting of outcomes
☐	☒	Estimates of effect sizes (e.g. Cohen's *d*, Pearson's *r*), indicating how they were calculated

Our web collection on statistics for biologists contains articles on many of the points above.

Software and code

Policy information about availability of computer code

Data collection	Chlorophyll content meter (CCM 200 plus Opti-Sciences, commercial), solar irradiance and spectrum (ILT900-R Spectroradiometer, InternationalLight Technologies, commercial), mRNA samples concentration and quality (2000 Nanodrop, NanoDrop Technologies and Agilent 2100 BioAnalyzer, Agilent Technologies, Qubit™, commercial), mRNA sequencing (Illumina NovaSeq 6000, commercial), GC-MS metabolomics (ChromaTOF, commercial), hormonomics (Agilent MassHunter, Agilent Technologies, commercial), spectrophotometer (SpectraMax 190, Molecular Devices, commercial).
Data analysis	Chlorophyll curve fitting and data visualization (OriginLab version 2021b, commercial), weather data (SHMI, TFE, open source), mRNA-seq data processing steps FastQC v0.10.1, SortMeRNA v2.1b, Trimmomatic v0.32 and Salmon v0.14.2. Differential gene expression DESeq2 v1.16.1 (R version 4.0.0, R-Core-Team 2015, GitHub repository https://doi.org/10.5281/zenodo.5906743), GC-MS metabolomics (data processing, In-house software, Swedish Metabolomics Centre, SMC_RDA [will be published later, demo version provided for the review purpose]), metabolite libraries (Golm metabolome database, open, NIST, commercial), statistics (MetaboAnalyst version 5.0, open), PCA and OPLS-DA (Simca P+ version 15, Umetrics, commercial), Gene Ontology (GO) term enrichment analyses (PlantGenIE, open), Kyoto Encyclopedia of Genes and Genomes (KEGG) pathway enrichment analysis (g:Profiler, update 2019, open), weighed gene coexpression network (R package WGCNA version 1.69, open), network visualization (Cytoscape, version 3.8.0, open), GO term network (ClueGO version 2.5.7, CluePedia version 1.5.7, open).

For manuscripts utilizing custom algorithms or software that are central to the research but not yet described in published literature, software must be made available to editors and reviewers. We strongly encourage code deposition in a community repository (e.g. GitHub). See the Nature Portfolio guidelines for submitting code & software for further information.

Data

Policy information about availability of data

All manuscripts must include a data availability statement. This statement should provide the following information, where applicable:

- Accession codes, unique identifiers, or web links for publicly available datasets
- A description of any restrictions on data availability
- For clinical datasets or third party data, please ensure that the statement adheres to our policy

Transcriptome data are available in the European Nucleotide Archive (ENA) under the accession number: PRJEB51801 (https://www.ebi.ac.uk/ena/browser/view/PRJEB51801). Transcriptome data for 201 in 2011 are available in Gene Expression Omnibus (GEO) repository with accession number GSE86960 (https://www.ncbi.nlm.nih.gov/geo/query/acc.cgi?acc=GSE86960). Other data generated in this study are available in the Supplementary data sets and Source data files provided with this paper.

Poplar (P. trichocarpa) gene lists were obtained from the Leaf Senescence Database (LSD 3.0, https://bigd.big.ac.cn/lsd/poplar.php) and from the Supplementary materials in Lu et al., 22.

Research involving human participants, their data, or biological material

Policy information about studies with human participants or human data. See also policy information about sex, gender (identity/presentation), and sexual orientation and race, ethnicity and racism.

Reporting on sex and gender	n/a
Reporting on race, ethnicity, or other socially relevant groupings	n/a
Population characteristics	n/a
Recruitment	n/a
Ethics oversight	n/a

Note that full information on the approval of the study protocol must also be provided in the manuscript.

Field-specific reporting

Please select the one below that is the best fit for your research. If you are not sure, read the appropriate sections before making your selection.

☐ Life sciences ☐ Behavioural & social sciences ☒ Ecological, evolutionary & environmental sciences

For a reference copy of the document with all sections, see nature.com/documents/nr-reporting-summary-flat.pdf

Ecological, evolutionary & environmental sciences study design

All studies must disclose on these points even when the disclosure is negative.

Study description

We performed transcriptomic (mRNA levels, gene expression), metabolomic (individual metabolites) and spectrophotometric analyses (reactive oxygen species-ROS, ROS-scavenging capacity [metabolic and enzyme activities], starch and total metabolite pools) of aspen leaf samples in autumn over two study years. Transcriptomics analyses: DEseq2 was used to identify differentially expressed genes between three genotypes (1, 48, 81) and nine time points in 2018 (225-264 day of the year), n=3 in each genotype and time point, except n=2 in genotype 48 on 237 doy (outlier sample omitted due to small library size, and samples from 270 doy that were from yellow leaves omitted due to their large contribution to the overall variation in the data). The effects of genotypes, time points and their interaction were tested in different ways, by Wald's contrast and by Likelihood ratio tests (LRT). The effect of time on gene expression was tested by comparing consecutive time points and by comparing time points to the first time point (225 doy). The results of all the different tests are presented in Supplementary Data 1. An overview of the mRNA data processing steps and parameters are available in the GitHub repository (https://doi.org/10.5281/zenodo.5906743).

Metabolomics (GC-MS- gas chromatography, mass spectrometry) and hormonomics (LC-MS, liquid chromatography-mass spectrometry): five genotypes (1, 33, 48, 81, 96) and eleven time points during autumn 2018 (217-270 doy). Metabolomics (GC-MS): One genotype 201 and twelve time points during autumn 2011. In 2011, n= 1 in each time point, in 2018, n=3 unless mentioned otherwise (n for each analyte in each genotype and particular time point is mentioned in the Supplementary Data 14). If the metabolite level was below the limit of detection (<LOD) the value is missing which affects the n for the particular analyte in the particular time point (no missing value imputation applied). Full factorial two-way ANOVA was used with false discovery rate (FDR) correction to test the effects of genotype, time and their interaction on the levels of GC-MS metabolites, phytohormones, starch, metabolite pools, ROS and ROS-scavenging capacity. The results (F, raw P-values, FDR-adjusted P-values) are presented in Supplementary Data 14 Table c.

The results of transcriptomics and metabolomics analyses were integrated in network analyses (weighed gene co-expression network analysis, WGCNA). WGCNA was applied to identify gene modules and their signature expression patterns (eigengenes). Then we studied the correlations between genes/eigengenes and weather parameters and metabolite markers to identify factors behind the

transcriptome responses in aspen leaves in autumn. WGCNA results are provided in Supplementary Data 3-13.

Research sample	All of the collected data were from the leaves of mature European aspen (Populus tremula Michx.) trees growing in field conditions (university campus or in the common garden). Five Swedish aspen (SwAsp) genotypes were scored for senescence onset by measuring chlorophyll levels twice a week from five leaves during autumn from 6th of August (218 DOY) until 9th of October (282 DOY). Genotype 201 included in the study is local situated at the university campus. The clonal replicates of 201 are included in the Umea aspen collection (UmAsp), they were 6 years old in 2018 when they were scored for senescence onset in the common garden in Sävar, Umeå, Sweden. The SwAsp collection was grown in the same common garden in Sävar, and all of the genotypes in the collection were scored for senescence onset during years 2011 (Michelson eta l. 2018) and the selected genotypes in 2018 (current manuscript). In 2018, they were sampled for transcriptomics and metabolomic analyses and they were 14 years old. Therefore, the study represents a local genotype and Swedish aspen genotypes originating from south, central and north of Sweden. We re-analysed transcriptomic data from 201 in 2011 (re-aligned with Salmon using the new version of P. tremula genome, v2.2). The original raw data has been deposited to Gene Expression Omnibus (GEO) repository with accession number GSE86960 and results for cytokinin levels and related gene expression published in Edlund et al. 2017. Published cytokinin levels were integrated with transcriptomics and metabolomics data in the co-expression network analyses.
Sampling strategy	We sampled healthy-looking short shoot leaves twice a week from 6th of August (218 DOY) until 9th of October (282 DOY). This frequent sampling is possible with mature trees where the removal of few leaves at a time has minimal effect on the overall leaf biomass in the canopy. Leaves were collected by cutting the short shoots with a telescopic tree pruner. Short shoot leaves are around the same age since they flush around the same time in spring. Five leaves were collected from the middle of the canopy level from four sides of the tree to get representative material for the tree. The trees were sampled at random and the sampling was always performed at noon and finished within one hour (12 am - 1 pm) to minimize the effect of the sampling time. Leaves were pooled, wrapped in aluminium foil and frozen in liquid nitrogen, and it was performed as fast as possible within a couple of minutes. Samples were collected from three individual replicate trees per genotype in each time point, unless there were no leaves present anymore in late autumn. Genotypes for this study were selected based on their senescence phenotypes published earlier (Michelson et al. 2018). We chose genotypes so that they would display large variation in their senescence timing in autumn and a large latitudinal range for their origin. Genotypes had to have at least three healthy-looking individual replicate trees growing in the common garden, which is typically considered as the minimum number of biological replicates. The increasing number of replicate trees would have also increased the time taken by the sampling.
Data collection	Two people performed the sampling and chlorophyll measurements in the field (JL with KMR or NF). Each measurement took only a few seconds to perform with a chlorophyll content meter and the instrument calculated the average of the measurements from five leaves (CCM 200 plus, Opti-Sciences). The time-dependent curves of chlorophyll content indices were then fitted by PB and JL using OriginLab software to estimate the senescence onset. Transcriptomics analyses were performed in SciLife lab in Stockholm, Sweden, with Illumina NovaSeq 6000 platform and the downstream data processing were performed by JL and ND. Metabolomics analyses with GC-MS were performed by JL and hormonomics analyses with LC-MS by JS and ON. Data collections were performed with the commercial softwares (LECO ChromaTOF and Agilent MassHunter). Spectrophotometric assays were performed by JL and NF and the data were recorded with a microplate reader (SpectraMax 190, Molecular Devices).
Timing and spatial scale	In 2011, data and samples were collected as described in Edlund et al. 2017 from July to late September. In 2018, data and samples were collected from 6th Aug to 9th Oct. i.e. the data collection and sampling were performed in three to four day intervals.
Data exclusions	Transcriptomics 2018: In most of the cases, n=3 in each genotype per time point, except n=2 in one time point 237 doy in genotype 48. One sample was omitted as it was an outlier with a small library size. WGCNA was performed with 76 samples, outlier samples were excluded based on hierarchical clustering (Euclidean distance). GC-MS Metabolomics, hormonomics and spectrophotometric assays 2018: Since most of the leaves in genotype 96 were abscised on 270 doy, the sampling for that genotype was omitted. In most of the cases, n=3. However, some samples were omitted due to the low abundance of internal standards (GC-MS) or in some cases the levels of analytes were below the limit of detection (<LOD) which means that the values are missing. The number of replicates (n) in each genotype and time point is specified in Supplementary Data 14 Table b.
Reproducibility	Our experiment is performed in the natural field conditions, and thus the conditions outdoors cannot be reproduced as such. Therefore, our approach in this manuscript was to compare the results obtained one year with multiple genotypes with another study year and another genotype to confirm that the conclusions held true. In addition, we are currently performing senescence experiments with aspen genotypes indoors in controlled and semi-controlled conditions.
Randomization	Sampling and measurements for individual replicate trees were randomized each time point. RNA and metabolite extractions and the running order were also randomized.
Blinding	Samples were given only a number, thus the genotype and time point were not given to prevent any bias.

Did the study involve field work? ☒ Yes ☐ No

Field work, collection and transport

Field conditions	The field sites are maintained by the Skogforsk (Forestry Research Institute of Sweden) in Sävar, Umeå, Sweden. The trees in the common garden have been appropriate marked and genotyped (genomic DNA sequenced). Weather parameters (temperature and humidity) in the field site have been recorded with data loggers, but for this study we chose to use several other weather parameters obtained from the Swedish Meteorological and Hydrological Institute (SHMI) and the weather station located at the university campus (TFE). Environmental parameters considered in this study were air temperature, relative air humidity, air vapour pressure deficit, air pressure, precipitation, sunshine hours, solar radiation, photoperiod, twilight period and night period. Weather conditions in the field during the sampling ranged from sunny to foggy and full overcast days. Sampling was omitted on rainy days.

Location	The common garden (63.9°N) where the sampling was performed is approx. 17 km from the Umeå university. The genotype 201 grows at the university campus (63.8°N).
Access & import/export	Liquid nitrogen container was transported to the field site to fast-freeze the samples and to store them during the transport back to the university.
Disturbance	This study did not cause any disturbance. It was conducted in a designated site for forest research.

Reporting for specific materials, systems and methods

We require information from authors about some types of materials, experimental systems and methods used in many studies. Here, indicate whether each material, system or method listed is relevant to your study. If you are not sure if a list item applies to your research, read the appropriate section before selecting a response.

Materials & experimental systems

n/a	Involved in the study	
☒	☐	Antibodies
☒	☐	Eukaryotic cell lines
☒	☐	Palaeontology and archaeology
☒	☐	Animals and other organisms
☒	☐	Clinical data
☒	☐	Dual use research of concern
☐	☒	Plants

Methods

n/a	Involved in the study	
☒	☐	ChIP-seq
☒	☐	Flow cytometry
☒	☐	MRI-based neuroimaging
