# Supplementary material for: Salicylic acid metabolism and signalling coordinate senescence initiation in aspen in nature
Source: Nat Commun. 2023 Jul 18;14:4288. doi: 10.1038/s41467-023-39564-5 (PMC10354028; doi:10.1038/s41467-023-39564-5)
Supplement: Supplementary file 4 — Description of Additional Supplementary Files [file 41467_2023_39564_MOESM4_ESM.pdf]

Description of Additional Supplementary Files

File Name: Supplementary Data 1
Description: Differentially expressed (DE) genes in three SwAsp genotypes in autumn 2018.

File Name: Supplementary Data 2
Description: Up- and down-regulated genes during autumn in aspen and in *Populus* spp.

File Name: Supplementary Data 3
Description: Weighted gene co-expression analysis (WGCNA) module membership in three SwAsp genotypes in autumn 2018.

File Name: Supplementary Data 4
Description: Weighted gene co-expression analysis (WGCNA) results in three SwAsp genotypes in autumn 2018.

File Name: Supplementary Data 5
Description: Weighted gene co-expression analysis (WGCNA) module membership in genotype I201 (campus tree) in autumn 2011.

File Name: Supplementary Data 6
Description: Weighted gene co-expression network analysis (WGCNA) results in genotype I201 in autumn 2011.

File Name: Supplementary Data 7
Description: Correlation between gene expression and environmental parameters in three SwAsp genotypes in autumn 2018.

File Name: Supplementary Data 8
Description: Correlation between gene expression and phytohormones in three SwAsp genotypes in autumn 2018.

File Name: Supplementary Data 9
Description: Correlation between gene expression, chlorophyll content index (CCI) and metabolic markers in three SwAsp genotypes in autumn 2018.

File Name: Supplementary Data 10
Description: Correlation between gene expression and GC-MS metabolites in three SwAsp genotypes in autumn 2018.

File Name: Supplementary Data 11
Description: Correlation between gene expression and environmental parameters in genotype I201 in autumn 2011.

File Name: Supplementary Data 12
Description: Correlation between gene expression and cytokinins (CK) in genotype I201 in autumn 2011.

File Name: Supplementary Data 13

Description: Correlation between gene expression, chlorophyll content index (CCI), GC-MS metabolites and other metabolic markers in genotype I201 in autumn 2011.

File Name: Supplementary Data 14
Description: Details and statistics of phytohormone and metabolomic marker levels and metabolite ratios in five SwAsp genotypes in autumn 2018 and in genotype I201 in autumn 2011.

File Name: Supplementary Data 15
Description: Relationships between gene expression, phytohormone levels, environmental parameters and chlorophyll content index (CCI).

File Name: Supplementary Data 16
Description: Biomarker analysis for senescence-associated metabolic markers and predicting the senescence onset date.
